# Effects of neonicotinoids and fipronil on non-target invertebrates

**DOI:** 10.1007/s11356-014-3471-x

**Published:** 2014-09-17

**Authors:** L. W. Pisa, V. Amaral-Rogers, L. P. Belzunces, J. M. Bonmatin, C. A. Downs, D. Goulson, D. P. Kreutzweiser, C. Krupke, M. Liess, M. McField, C. A. Morrissey, D. A. Noome, J. Settele, N. Simon-Delso, J. D. Stark, J. P. Van der Sluijs, H. Van Dyck, M. Wiemers

**Affiliations:** 1 Environmental Sciences, Copernicus Institute, Utrecht University, Heidelberglaan 2, 3584 CS Utrecht, The Netherlands; 2 Buglife, Bug House, Ham Lane, Orton Waterville, Peterborough, PE2 5UU UK; 3 Laboratoire de Toxicologie Environnementale, INRA, UR 406 Abeilles & Environnement, Site Agroparc, 84000 Avignon, France; 4 Centre de Biophysique Moléculaire, UPR 4301 CNRS, affiliated to Orléans University and to INSERM, 45071 Orléans cedex 02, France; 5 Haereticus Environmental Laboratory, P.O. Box 92, Clifford, VA 24533 USA; 6 School of Life Sciences, University of Sussex, Sussex, BN1 9RH UK; 7 Canadian Forest Service, Natural Resources Canada, 1219 Queen Street East, Sault Ste Marie, ON P6A 2E5 Canada; 8 Department of Entomology, Purdue University, West Lafayette, IN USA; 9 Department System-Ecotoxicology, Helmholtz Centre for Environmental Research, UFZ, Permoserstrasse 15, 04318 Leipzig, Germany; 10 Healthy Reefs for Healthy People Initiative, Smithsonian Institution, Belize City, Belize; 11 Department of Biology and School of Environment and Sustainability, University of Saskatchewan, 112 Science Place, Saskatoon, SK S7N 5E2 Canada; 12 Task Force on Systemic Pesticides, 46, Pertuis-du-Sault, 2000 Neuchâtel, Switzerland; 13 Kijani, Kasungu National Park, Private Bag 151, Lilongwe, Malawi; 14 Department of Community Ecology, Helmholtz-Centre for Environmental Research, UFZ, Theodor-Lieser-Str. 4, 06120 Halle, Germany; 15 German Centre for Integrative Biodiversity Research (iDiv), Halle-Jena-Leipzig, Deutscher Platz 5e, 04103 Leipzig, Germany; 16 Beekeeping Research and Information Centre (CARI), Place Croix du Sud 4, 1348 Louvain-la-Neuve, Belgium; 17 Puyallup Research and Extension Centre, Washington State University, Puyallup, WA 98371 USA; 18 Centre for the Study of the Sciences and the Humanities, University of Bergen, Postboks 7805, 5020 Bergen, Norway; 19 Behavioural Ecology and Conservation Group, Biodiversity Research Centre, Earth and Life Institute, Université Catholique de Louvain (UCL), Croix du Sud 4-5, bte L7.07.04, 1348 Louvain-la-Neuve, Belgium

**Keywords:** Pesticides, Neonicotinoids, Fipronil, Non-target species, Invertebrates, Honeybee, Earthworms, Butterflies, Freshwater habitat, Marine habitat

## Abstract

We assessed the state of knowledge regarding the effects of large-scale pollution with neonicotinoid insecticides and fipronil on non-target invertebrate species of terrestrial, freshwater and marine environments. A large section of the assessment is dedicated to the state of knowledge on sublethal effects on honeybees (*Apis mellifera*) because this important pollinator is the most studied non-target invertebrate species. Lepidoptera (butterflies and moths), Lumbricidae (earthworms), Apoidae sensu lato (bumblebees, solitary bees) and the section “other invertebrates” review available studies on the other terrestrial species. The sections on freshwater and marine species are rather short as little is known so far about the impact of neonicotinoid insecticides and fipronil on the diverse invertebrate fauna of these widely exposed habitats. For terrestrial and aquatic invertebrate species, the known effects of neonicotinoid pesticides and fipronil are described ranging from organismal toxicology and behavioural effects to population-level effects. For earthworms, freshwater and marine species, the relation of findings to regulatory risk assessment is described. Neonicotinoid insecticides exhibit very high toxicity to a wide range of invertebrates, particularly insects, and field-realistic exposure is likely to result in both lethal and a broad range of important sublethal impacts. There is a major knowledge gap regarding impacts on the grand majority of invertebrates, many of which perform essential roles enabling healthy ecosystem functioning. The data on the few non-target species on which field tests have been performed are limited by major flaws in the outdated test protocols. Despite large knowledge gaps and uncertainties, enough knowledge exists to conclude that existing levels of pollution with neonicotinoids and fipronil resulting from presently authorized uses frequently exceed the lowest observed adverse effect concentrations and are thus likely to have large-scale and wide ranging negative biological and ecological impacts on a wide range of non-target invertebrates in terrestrial, aquatic, marine and benthic habitats.

## Introduction

Neonicotinoids and fipronil are relatively new, widely used, systemic compounds designed as plant protection products to kill insects which cause damage to crops. They are also used in veterinary medicine to control parasites such as fleas, ticks and worms on domesticated animals and as pesticides to control non-agricultural pests. Other papers in this special issue have shown that neonicotinoid insecticides and fipronil are presently used on a very large scale (e.g. Simon-Delso et al. 2014, this issue), are highly persistent in soils, tend to accumulate in soils and sediments, have a high runoff and leaching potential to surface and groundwater and have been detected frequently in the global environment (Bonmatin et al. 2014, this issue). Effects of exposure to the large-scale pollution with these neurotoxic chemicals on non-target insects and possibly other invertebrates can be expected as identified for other insecticides. However, for the majority of insect and other invertebrate species that are likely to be exposed to neonicotinoids and fipronil in agricultural or (semi)natural ecosystems, no or very little information is available about the impact of these pesticides on their biology. Here we assess the present state of knowledge on effects on terrestrial and aquatic invertebrates.

## Terrestrial invertebrates

### Honeybees

Many studies have focused on investigating the effects of neonicotinoids and fipronil on honeybees (*Apis mellifera*). Apart from its cultural and honey production value, the honeybee is the most tractable pollinator species and critical for the production of many of the world’s most important crops (Klein et al. 2007; Breeze et al. 2011). Losses of honeybees are generally measured as winter loss on national to regional level, and indications are that honeybee populations undergo high losses in many parts of the world (Oldroyd 2007; Stokstad 2007; van Engelsdorp and Meixner 2010; Van der Zee et al. 2012a, b).

No single cause for high losses has been identified, and high losses are associated with multiple factors including pesticides, habitat loss, pathogens, parasites and environmental factors (Decourtye et al. 2010; Mani et al. 2010; Neumann and Carreck 2010; Kluser et al. 2011). Apart from direct biotic and abiotic factors, changes in honeybee populations also depend on the economic value of honeybees and thus on human effort (Aizen and Harder 2009; Mani et al. 2010). Neonicotinoids are among the most used insecticides worldwide and are thus prime targets for investigating possible relationships with high honeybee losses.

#### Acute and chronic lethal toxicity to honeybees

Neonicotinoids and fipronil show high acute toxicity to honeybees (Table 1). The neonicotinoid family includes imidacloprid, clothianidin and thiamethoxam (the latter is metabolized to clothianidin in the plant and in the insect). Imidacloprid, clothianidin and thiamethoxam belong to the nitro-containing neonicotinoids, a group that is generally more toxic than the cyano-containing neonicotinoids, which includes acetamiprid and thiacloprid. Although neonicotinoids are applied as foliar insecticides with possible direct exposure risks to honeybees, a large part of neonicotinoid use consists of seed coating or root drench application. Fipronil belongs to the phenylpyrazole family of pesticides and, like the neonicotinoids, has systemic properties (Simon-Delso et al. 2014).

**Table 1:** Toxicity of insecticides to honeybees, compared to DDT. Dose used is given in gram per hectare, median lethal dose (LD_50_) is given in nanogram per bee. The final column expresses toxicity relative to DDT (DDT is 1). Source: Bonmatin

Pesticide	®Example	Main use	Typical dose (g/ha)	Acute LD_50_ (ng/bee)	Ratio of LD_50_ as compared to DDT
DDT	Dinocide	Insecticide	200–600	27.000	1
Thiacloprid	Proteus	Insecticide	62.5	12.600	2.1
Amitraz	Apivar	Acaricide	–	12.000	2.3
Acetamiprid	Supreme	Insecticide	30–150	7.100	3.8
Coumaphos	Perizin	Acaricide	–	3.000	9
Methiocarb	Mesurol	Insecticide	150–2.200	230	117
Tau-fluvalinate	Apistan	Acaricide	–	200	135
Carbofuran	Curater	Insecticide	600	160	169
Λ-cyhalotrin	Karate	Insecticide	150	38	711
Thiametoxam	Cruiser	Insecticide	69	5	5.400
Fipronil	Regent	Insecticide	50	4.2	6.475
Imidacloprid	Gaucho	Insecticide	75	3.7	7.297
Clothianidin	Poncho	Insecticide	50	2.5	10.800
Deltamethrin	Decis	Insecticide	7.5	2.5	10.800

Given that neonicotinoids and fipronil act systemically in plants, oral lethal doses for honeybees have been extensively studied for these compounds. Unlike many older classes of insecticides, neonicotinoids may be more toxic when ingested (Suchail et al. 2001; Iwasa et al. 2004). The level of neonicotinoids and fipronil that honeybees are exposed to in the nectar and pollen of treated plants varies greatly, although there are trends based upon application method. Generally, soil drenches and foliar application result in higher concentrations of the active compounds in plants than seed treatments, with the latter application used in large, annual cropping systems like grain crops, cotton and oilseed crops.

In practice, the honeybee lethal dose 50 (LD_50_) for these pesticides varies for a wide range of biotic and abiotic conditions. The LD_50_ of imidacloprid, for example, has shown values between 3.7 and 40.9, 40 and 60, 49 and 102 and 490 ng/bee (Nauen et al. 2001; Schmuck et al. 2001; Suchail et al. 2001; DEFRA 2007, 2009). This variation, of a factor 100 (5–500 ng/bee), has been observed not only between colonies but also among bees taken from a single colony. A major component of this observed variation likely stems from the discrepancy in the contact and oral toxicities of these compounds, with contact lethal doses generally being higher than oral lethal doses. However, contact with the floral parts is frequent when the bees visit flowers, and this is different from the topical application used in laboratory conditions.

Other sources of variability may be attributed to differences in environmental conditions during testing as well as any inherent differences in the condition of the tested bees themselves. For example, data have shown that measured LD_50_ values for bees vary with temperature (Medrzycki et al. 2011), the age of bees (Schmuck 2004; Medrzycki et al. 2011), the honeybee subspecies tested (Suchail et al. 2000), the pattern of exposure (Illarionov 1991; Belzunces 2006) and prior exposure of bees to pesticides (Belzunces 2006). Given the large variability of honeybee toxicity data, it has been suggested that LD_50_ values should only be used to compare levels of toxicity among pesticides rather than drawing conclusions about the risk of mortality posed to honeybees via environmental exposure to pesticides (Belzunces 2006).

Oral subchronic exposure to imidacloprid and six of its metabolites induced a high toxicity at concentrations of 0.1, 1 and 10 ppb (part per billion) or ng/g, whereas the metabolites olefin-imidacloprid and 5-OH-imidacloprid were toxic in acute exposure. The main feature of subchronic toxicity is the absence of a clear dose–effect relationship that can account for a maximum effect of the lowest concentration due to the existence of multiple molecular targets, as has been demonstrated in the honeybee (Déglise et al. 2002; Thany et al. 2003; Thany and Gauthier 2005; Barbara et al. 2008; Gauthier 2010; Dupuis et al. 2011; Bordereau-Dubois et al. 2012). The absence of clear dose–effect relationships has also been observed in other studies, at higher concentrations (Schmuck 2004).

Existence of non-monotonic dose–response relations implies that some chemicals, including neonicotinoids, have unexpected and potent effects at (very) low doses. These non-linear and often non-intuitive patterns are due to the complex interplay of receptor binding and gene reprogramming effects of such substances and can generate unexpected dose–response relationships, many of which are still being mapped out (Fagin 2012; Charpentier et al. 2014). This poses major challenges to risk assessment based on the classical log-probit model.

As previously reviewed by van der Sluijs et al. (2013), there are no standardised protocols for measuring chronic lethal effects. In traditional risk assessment of pesticides, they are usually expressed in three ways: LD_50_: the dose at which 50 % of the exposed honeybees die (usually within a 10 day time span); no observed effect concentration (NOEC): the highest concentration of a pesticide producing no observed effect; and lowest observed effect concentration (LOEC): the lowest concentration of a pesticide producing an observed effect.

For imidacloprid, including its neurotoxic metabolites, lethal toxicity can increase up to 100,000 times compared to acute toxicity when the exposure is extended in time (Suchail et al. 2001). There has been some controversy on the findings of that study, which are discussed in detail by Maxim and Van der Sluijs (2007, 2013). However, the key finding that exposure time amplifies the toxicity of imidacloprid is consistent with later findings, implying that the standard 10 day chronic toxicity test for bees is far too short for testing neonicotinoids and fipronil, given their persistence and hence the likely chronic exposure of bees under field conditions. Indeed, honeybees fed with 10^−1^ of the LC_50_ of thiamethoxam showed a 41.2 % reduction of life span (Oliveira et al. 2013). Recent studies have shown that chronic toxicity of neonicotinoids can more adequately be expressed by time to 50 % mortality instead of by the 10 day LD_50_ (Sánchez-Bayo 2009; Maus and Nauen 2010; Tennekes 2010; Tennekes 2011; Tennekes and Sánchez-Bayo 2012; Mason et al. 2013; Rondeau et al. 2014). There is a linear relation between the logarithm of the daily dose and the logarithm of the time to 50 % mortality (Tennekes 2010, 2011; Tennekes and Sánchez-Bayo 2012; Tennekes and Sánchez-Bayo 2013; Rondeau et al. 2014). Sanchez-Bayo and Goka (2014) demonstrated that field-realistic residues of neonicotinoid insecticides in pollen pose high risk to honeybees and bumblebees, whilst in the field synergisms with ergosterol inhibiting fungicides will further amplify these risks. They found that imidacloprid poses the highest risk to bumblebees (31.8–49 % probability to reach the median lethal cumulative dose after 2 days feeding on field-realistic dose in pollen) and thiamethoxam the highest risk to honeybees (3.7–29.6 % probability to reach median lethal cumulative dose). In experiments with honeybee colonies, similar, long-term chronic effects have been found with typical times of 80–120 days for 1 ppm dinotefuran and 400 ppb clothianidin (Yamada et al. 2012). Note that these studies used concentrations that are on the uppermost limit of the currently reported ranges of concentrations found in pollen and nectar in the field. However, such data are sparse and limited to a few crops only, so it cannot yet be concluded whether such concentrations are rare or common in the field—the question of “field-relevant dose” is not yet fully resolved, and it is likely that there is a wide range in these values over space and time (Van der Sluijs et al. 2013).

Field and laboratory studies attempting to test field-realistic lethal doses have shown variable, often conflicting, results. In one study, chronic oral and contact exposure during 10–11 days to 1 μg/bee of acetamiprid and 1,000 μg/bee of thiamethoxam caused no significant worker mortality (Aliouane et al. 2009). Conversely, laboratory studies using imidacloprid showed high worker mortality when honeybees consumed contaminated pollen (40 ppb) (Decourtye et al. 2003, 2005) and contaminated sugar syrup (0.1, 1.0 and 10 ppb) (Suchail et al. 2001). These results were contrary to those of field studies performed by Schmuck et al. (2001), who reported no increased worker mortality when colonies were exposed to sunflower nectar contaminated with imidacloprid at rates from 2.0 to 20 μg/kg. Faucon et al. (2005) also found no worker mortality in a field study of honeybees fed imidacloprid in sugar syrup. A meta-analysis by Cresswell (2011) concluded that oral exposure to imidacloprid at realistic field concentrations did not result in worker mortality, although a subsequent study by Yamada et al. (2012) feeding a range of dinotefuran (1–10 ppm) and clothianidin (0.4–4 ppm) concentrations demonstrated colony failure within 104 days in each case, suggesting that detection of colony-level effects may require longer post**-**exposure observation.

Field studies to investigate the exposure of bees to pesticides face major difficulties. For the analysis of very low concentrations of compounds present in pollen, nectar, bees or other matrices, appropriate methods that meet validity criteria of quantitative analysis have to be developed. Pilling et al. (2013) exposed bees to thiamethoxam-treated maize and oilseed rape but were not able to quantify concentrations lower than 1 ppb, although this may be a result of the authors using a lower seed treatment application than is used in normal agricultural practice. Even though both treatment and control colonies experienced relatively high losses (mostly queens laying only drone brood) and the authors were unable to undertake any statistical analysis due to a lack of replication, they wrongly concluded that there is a low risk to honeybees from exposure to treated maize and oilseed rape.

Also, in terms of activity and feeding behaviour, bees might not be foraging on treated crops in (exactly) the same way as they would do on untreated crops (Colin et al. 2004). Furthermore, comparison of treated and control areas can be totally flawed because control fields might not be “clean” but treated with other pesticides, including insecticides. The recent study of Pilling and co-workers on thiamethoxam (Pilling et al. 2013) is illustrative for this case as it did not provide information about the treatment status of the control plots.

For mass-dying of bees in spring near corn fields during sowing of neonicotinoid-treated seeds, there now is a one to one proven causal link. Acute intoxication occurs through exposure to the dust cloud around the pneumatic sowing machines during foraging flights to adjacent forests (providing honeydew) or nearby flowering fields (Apenet 2010; Girolami et al. 2012; Tapparo et al. 2012; Krupke et al. 2012; Pochi et al. 2012; Tapparo et al. 2012). In these cases, dead bees have typically been found to have high levels of seed treatment neonicotinoids on, or in, their bodies. Such mass colony losses during corn sowing have been documented in Italy, Germany, Austria, Slovenia, the USA and Canada (Gross 2008; Krupke et al. 2012; Sgolastra et al. 2012; Tapparo et al. 2012). In response to the incidents, the adherence of the seed coating has been improved owing to better regulations, and an improved sowing technique has recently become compulsory throughout Europe (European Commission 2010). However, despite the deployment of air deflectors in the drilling machines and improved seed coating techniques, emissions are still substantial and the dust cloud remains acutely toxic to bees (Biocca et al. 2011; Marzaro et al. 2011; Girolami et al. 2012; Tapparo et al. 2012; Sgolastra et al. 2012).

Acute lethal effects of neonicotinoids dispersed as particles in the air seem to be promoted by high environmental humidity (Girolami et al. 2012). Honeybees also transport toxic dust particles on their bodies into the hive (Girolami et al. 2012). Sunny and warm days also seem to favour the dispersal of active substances (Greatti et al. 2003).

#### Sublethal effects on honeybees

##### Effects on activity, locomotion, metabolism and ontogenetic development

Imidacloprid, thiamethoxam and clothianidin have been shown to rapidly induce flight muscle paralysis in honeybees exposed to guttation drops containing these substances, resulting in the cessation of wing movements (Girolami et al. 2009). Imidacloprid further impairs the mobility of bees, as reflected by decreases in running and walking and increases in the time that exposed bees remain stationary (Medrzycki et al. 2003). However, when exposed to subchronic doses of neonicotinoids, decreases in locomotion were not observed in honeybees and bumblebees by Cresswell et al. (2012b).

Ontogenetic development is a crucial period that determines the physiological and functional integrity of adult individuals. Thus, in addition to the effects on adults, neonicotinoids may act on larval development with consequences for the adult stage. Adult honeybees exposed to imidacloprid during the larval stage exhibit impairment of olfactory associative behaviour (Yang et al. 2012). This could be due to altered neural development. Impairments in mushroom body development in the bee brain and the walking behaviour of honeybee workers have been observed in individuals exposed to imidacloprid during the larval period (Tomé et al. 2012). Effects on adult bees exposed during the larval stage could also be attributed to the induction of cell death by imidacloprid in larvae (Gregorc and Ellis 2011). In the early stages of adult life, after emergence, imidacloprid can disrupt the development of hypopharyngeal glands by decreasing the size of the acini and by increasing the expression of hsp70 and hsp90 (Smodis Skerl et al. 2009; Hatjina et al. 2013). Derecka et al. (2013) provided beehives in the field for 15 days with syrup tainted with 2 μg/l imidaclopid. They found that these levels of imidacloprid, at the low end of the field-realistic range, significantly impact energy metabolism in worker bee larvae.

Impacts of pesticides on metabolism may affect the detoxifying, intermediary and energetic metabolism pathways. Imidacloprid impairs brain metabolism in the honeybee which results in an increase of cytochrome oxidase in mushroom bodies (Decourtye et al. 2004a, b).

##### Effects on behaviour, learning and memory

Optimal function of the honeybee nervous system is critical to individual and colony functioning (Desneux et al. 2007; Thompson and Maus 2007). Increasing levels of research effort have been devoted to developing an improved understanding of how sublethal exposure to neonicotinoids and fipronil may affect the honeybee nervous system. There is evidence that sublethal exposure can affect learning, memory and orientation in honeybees.

Laboratory experiments administering a single dose of imidacloprid demonstrated that learning was altered (Guez et al. 2001; Lambin et al. 2001), and exposure to chronic sublethal doses has demonstrated that learning and foraging are impaired by imidacloprid and fipronil (Decourtye et al. 2003). Furthermore, thiamethoxam has been shown to decrease memory capacity (Aliouane et al. 2009). The methodologies and doses varied in these laboratory tests, but all used concentrations above 20 ppb; this is towards the upper end of concentrations found in most field situations. These concentrations would not be expected to be found in pollen or nectar following seed treatment applications, but have been found in cucurbit flowers following soil drench applications (Dively and Hooks 2010). Field experiments offer the potential for powerful tests; however, results have been mixed, and many studies focus on honeybee orientation to and from a feeding source. A study that trained honeybee foragers to a sugar syrup reward in a complex maze demonstrated that 38 % of bees found the food source following ingestion of 3 ng/bee of thiamethoxam, compared with 61 % in an unexposed control group (Decourtye and Devillers 2010). A series of studies training foragers to orient to a sugar feeder found that foragers were unable to return to the hive after ingesting imidacloprid at concentrations ranging from 100 to 1,000 ppb (Bortolotti et al. 2003; Ramirez-Romero et al. 2005; Yang et al. 2008). In contrast, other semi-field studies have shown no effects upon foraging or survivorship following exposure to canola, maize and sunflowers grown from neonicotinoid-treated seeds (Schmuck et al. 2001; Cutler and Scott-Dupree 2007; Nguyen et al. 2009). Possible explanations for these conflicting results may be that when given a range of foraging opportunities, honeybees may reduce foraging visits to food sources containing pesticides (Mayer and Lunden 1997; Colin et al. 2004), or that neonicotinoids do not have effects on colonies in the exposure regimes tested here.

Recently, Henry et al. (2012a, b) described the results of innovative field experiments using radio frequency identification (RFID) tags to determine the colony-level effects of orientation impairment upon foragers fed a sublethal dose of imidacloprid (1.42 ng in 20 μl of sucrose syrup). In two separate experiments, treated foragers failed to return to the colony at rates of 10.2 and 31.6 %, relative to untreated foragers feeding upon the same flowering plants. A higher risk of not returning was associated with the more difficult orientation tasks. Using these forager loss rates, the researchers modelled the colony-level effects and found significant, largely consistent deviations from normal colony growth rates, in some cases to levels that may put the colony at risk of collapse. A subsequent suggestion by Cresswell and Thompson (2012) to alter the simulation slightly to reflect the period when seed-treated crops are flowering demonstrated that the risk of collapse was no longer evident. However, a follow-up calculation by Henry et al. (2012a) using a larger dataset that incorporated a range of empirically derived colony growth estimates revealed even higher deviations from normal than the original work: a more serious negative outcome for colonies. The variable outcomes based upon model assumptions reflect uncertainties that have plagued honeybee researchers and further underscore the importance of ensuring that models are robust and represent a range of scenarios. The key contribution of this work was the demonstration that sublethal doses can impose a stressor (i.e. non-returning foragers) that can have significant negative outcomes on a colony level.

Learning and memory represent fundamental functions involved in the interaction of individuals with their environment and are critical in enabling bees to respond to the requirements of the colony throughout their life. Imidacloprid impairs learning and olfactory performance via both acute and chronic exposure pathways, and summer bees appear more sensitive than winter bees (Decourtye et al. 2003). These effects are observed not only in the laboratory but also in semi-field conditions, and bees do not recover after exposure ceases. Results obtained with acetamiprid and thiamethoxam showed that the action of neonicotinoids depends on the level/degree of exposure and cannot be generalized to structurally related compounds. Unlike contact exposure, oral exposure of acetamiprid resulted in an impairing of long-term retention of olfactory learning (El Hassani et al. 2008). Conversely, for thiamethoxam, subchronic exposure, but not acute exposure, elicited a decrease of olfactory memory and an impairment of learning performance (El Hassani et al. 2008; Aliouane et al. 2009).

Neonicotinoids have specific routes of metabolism in insects, particularly in the honeybee, that lead to complex influences on learning and memory processes. Imidacloprid and thiamethoxam are metabolized into toxic metabolites that may potentially bind to different honeybee nicotinic acetylcholine receptors (Nauen et al. 2001; Suchail et al. 2001, 2004a; Nauen et al. 2003; Ford and Casida 2006; Benzidane et al. 2010; Casida 2011). The metabolism of acetamiprid results in the appearance of different metabolites in the honeybee, among which 6-chloronicotinic acid is toxic in chronic exposure but not in acute exposure and remains stable for at least 72 h, especially in the head and the thorax (Suchail et al. 2001, 2004a; Brunet et al. 2005). Considering the presence of multiple active metabolites over time, it is very difficult to ascertain what steps of the memory process (acquisition, consolidation or retrieval) are affected by imidacloprid, acetamiprid, thiamethoxam or their metabolites.

Habituation may be defined as “a form of learning that consists in the gradual and relatively prolonged decrease of the intensity or the frequency of a response following the repeated or prolonged stimulus responsible for eliciting such a response” (Braun and Bicker 1992; Epstein et al. 2011a, b; Belzunces et al. 2012). Habituation can be regarded as an important adaptive behaviour because it allows individuals to minimize their response and, therefore, their energy investment, towards unimportant stimuli. The neonicotinoid imidacloprid alters patterns of habituation in honeybees following contact exposure to a sublethal dose (Guez et al. 2001; Lambin et al. 2001). Imidacloprid-induced changes in habituation appear to vary depending on the age of bees and time after exposure. Furthermore, these changes in habituation may be due to factors such as differential sensitivity of different nicotinic acetylcholine receptors (nAChRs) to imidacloprid (Déglise et al. 2002; Thany et al. 2003; Thany and Gauthier 2005; Barbara et al. 2008; Gauthier 2010; Dupuis et al. 2011; Bordereau-Dubois et al. 2012; Farooqui 2013), or the accumulation of imidacloprid metabolites like olefin and 5-hydroxy-imidacloprid, which can delay or accelerate habituation, respectively (Guez et al. 2001, 2003).

Olfaction and taste are very important physiological senses for honeybees (Detzel and Wink 1993; Giurfa 1993; Balderrama et al. 1996; Goulson et al. 2001; Reinhard et al. 2004; Gawleta et al. 2005; Couvillon et al. 2010; Maisonnasse et al. 2010; Kather et al. 2011). The effects of neonicotinoids on gustation can be explored by studying the modulation of the gustatory threshold, which can be defined as the lowest concentration of a sucrose solution applied to the antenna that triggers a feeding response. Different active compounds have been shown to induce dissimilar effects on gustation in honeybees. For example, fipronil increases the gustatory threshold of bees subjected to contact exposure (El Hassani et al. 2005). Whilst similar results were found for imidacloprid, acetamiprid decreases the threshold of bees that are exposed orally, but not topically (El Hassani et al. 2009). Thiamethoxam elicits a decrease in honeybee responsiveness to sucrose, and exposure to acetamiprid increases the responsiveness of honeybees to water regardless of exposure route (El Hassani et al. 2008; Aliouane et al. 2009).

The discrepancy in the effects observed could be explained in part by neonicotinoid metabolism that induced the appearance of toxic metabolites (Suchail et al. 2004a, b; Brunet et al. 2005) and by the existence of different nAChRs that are either sensitive and resistant to particular neonicotinoids (Déglise et al. 2002; Thany et al. 2003; Thany and Gauthier 2005; Barbara et al. 2008; Gauthier 2010; Dupuis et al. 2011; Bordereau-Dubois et al. 2012). Although it has been demonstrated in pollinating flies and in beetles, the repellent effect of imidacloprid and other neonicotinoids has not been investigated in the honeybee (Easton and Goulson 2013).

Accurate navigation is essential for efficient foraging and, hence, for colony health and survival. Neonicotinoids and fipronil may impair navigation in different ways. Sublethal exposure of honeybees to clothianidin and imidacloprid elicits a decrease in foraging activity and induces longer foraging flights (Schneider et al. 2012). Thiamethoxam induces high mortality by causing failure in the homing behaviour of foraging bees, leading to large losses of foragers from the colony (Henry et al. 2012a, b). Although this effect has been demonstrated for the pyrethroid deltamethrin for almost 20 years (Vandame et al. 1995), impacts on the homing behaviour of foraging bees continue to be left out of the assessment process for pesticide registration.

Proper foraging behaviour is essential for both individual bees and the colony as a whole because it determines the availability of food (stores) and, consequently, the survival of the colony. Exposure to imidacloprid, clothianidin and fipronil can lead to reductions in the proportion of active bees in the hive and, furthermore, initiate behaviours that can reduce the efficiency of foraging flights. For example, exposed individuals may spend longer periods of time at a food source, decrease the frequency of visits, increase the time between foraging trips, engage in longer foraging flights, reduce foraging distances, exhibit problems revisiting the same feeding site or exhibit reductions in visual learning capacities (Nielsen et al. 2000; Morandin and Winston 2003; Colin et al. 2004; Ramirez-Romero et al. 2005; Yang et al. 2008; Han et al. 2010; Schneider et al. 2012; Teeters et al. 2012). Fischer et al. (2014) exposed adult honeybees to sublethal doses of imidacloprid (7.5 and 11.25 ng/bee), clothianidin (2.5 ng/bee) and thiacloprid (1.25 μg/bee) and subsequently tracked the flight paths of individual bees with harmonic radar. The rate of successful return was significantly lower in treated bees, the probability of a correct turn at a salient landscape structure was reduced and less directed flights during homing flights were performed. These findings show that sublethal doses of these three neonicotinoids either block the retrieval of exploratory navigation memory or alter this form of navigation memory. Reproduction and colony development may be regarded as integrative endpoints for assessing the final impacts of pesticides on bees as both are a compulsory condition of social insect physiology.

Neonicotinoids such as thiacloprid, thiamethoxam and imidacloprid decrease brood production, larval eclosion, colony growth rate and the number of queens reared in bumblebees (Tasei et al. 2000; Mommaerts et al. 2010; Whitehorn et al. 2012). Studies suggest that the reduction in brood production may be associated with a reduction in pollen and sugar consumption by adult bees (Laycock et al. 2012a, b). The rearing of honeybees on brood comb containing high levels of pesticide residues results in delayed larval development and emergence and shortened adult longevity (Wu et al. 2011). Since the brood combs in the latter study contained five neonicotinoids at relatively high concentrations, it is difficult to ascribe the observed effects to any one pesticide, or pesticide class. An epidemiological study involving Hill’s criteria (minimal conditions that prove evidence of a causal relationship) revealed conflicting results on the involvement of dietary traces of neonicotinoids in the decline of honeybee populations (Cresswell et al. 2012a) and could not establish a causal link between observations of bee decline and neonicotinoid use rates.

#### Interaction with pathogens

Detrimental effects of pesticides might be increased in combination with other environmental stress agents (Mason et al. 2013). Specific pathogens and parasites are ancestral companions of (some) honeybee populations, and accidental movement of parasites and pathogens by man has exposed both honeybees and wild bees to non-native enemies to which they may have reduced resistance (e.g. Goulson 2003; Graystock et al. 2013a, b). Imidacloprid can act synergistically with the pathogen *Nosema* spp. by increasing *Nosema*-induced mortality (Alaux et al. 2010). It affects social immunity and so increases the number of *Nosema* spores in the guts of bees from imidacloprid-exposed colonies exposed in cage studies (Pettis et al. 2012). Sequential exposure to *Nosema ceranae* can sensitize bees to thiacloprid by eliciting potentiation that leads to high mortality rates, a feature shared with fipronil (Vidau et al. 2011; Aufauvre et al. 2012). Similarly, other experiments with fipronil and *N. ceranae* have demonstrated reciprocal sensitization (Aufauvre et al. 2012). Furthermore, exposure to pesticides during embryonic and post-embryonic development may alter the susceptibility of adult bees to pathogens. For example, adult honeybees reared in brood combs containing high levels of pesticide residues exhibit higher levels of infection by *N. ceranae* and higher levels of *Nosema* spores (Wu et al. 2012).

Di Prisco et al. (2013) demonstrated that clothianidin negatively modulates nuclear factor kappa-light-chain-enhancer of activated B cells (NF-κB, a protein involved in DNA transcription) immune signaling in insects and adversely affects honeybee antiviral defences controlled by this transcription factor. They identified a negative modulator of NF-κB activation specific for insects. Exposure to clothianidin, by enhancing the transcription of the gene encoding this inhibitor, reduces immune defences and promotes the replication of the deformed wing virus present in honeybees. Similar immunosuppression was found to be induced by imidacloprid. The occurrence of this insecticide-induced viral proliferation at sublethal doses that are well within field-realistic concentrations suggests that the studied neonicotinoids are likely to have a negative effect under field conditions.

#### Synergistic effects with other pesticides

In agricultural ecosystems, honeybees are seldom exposed to only a single pesticide. Combined exposures could be of high concern because they can elicit synergies and potentiations. For example, thiacloprid acts synergistically with ergosterol biosynthesis inhibitor (EBI) fungicides in bees exposed in laboratory conditions but not in tunnel conditions (Schmuck et al. 2003).

Analyses of honeybees and colony contents indicate that honeybees are indeed frequently exposed to multiple pesticides simultaneously (Mullin et al. 2010; Krupke et al. 2012; Paradis et al. 2013). However, the study of pesticide mixtures can be challenging (Lydy et al. 2004), and there is a paucity of information in the literature regarding the mixtures encountered by honeybees. Triazole fungicides have been found in pollen collected from colonies (Krupke et al. 2012) and have been shown to synergize toxicity of some neonicotinoids (thiacloprid and acetamiprid) up to 559-fold in the laboratory, although the same results have not been shown in semi-field studies (Schmuck et al. 2003). Piperonyl butoxide also has been found in pollen and has been shown to synergize toxicity of some neonicotinoids (thiacloprid and acetamiprid) up to 244-fold in the laboratory (Iwasa et al. 2004). Despite the challenges associated with this type of research, this is a clear research gap that should be addressed in the future, given that honeybees rarely encounter only a single pesticide during foraging and/or in the hive.

### Toxicity to bumblebees and solitary bees

Bumblebees (genus *Bombus*) are primitive social bees. Colonies start from overwintering queens, build up to a few hundred adult workers and break down when new queens and males are produced. A small number of bumblebee species are commercially reared for pollination, but many of the non-managed bumblebees also contribute substantially to crop pollination (Chagnon et al. 1993; Bosch and Kemp 2006; Greenleaf and Kremen 2006; Goulson 2010). Solitary bees that are also commonly managed in agricultural settings include the alfalfa leafcutter bee (*Megachile rotundata*), alkali bees (*Nomia melanderi*), blue orchard bees (*Osmia lignaria*) and Japanese horn-faced bees (*Osmia cornifrons*). *M. rotundata* is the major pollinator of alfalfa, which is grown as a high value livestock feed in North America. It is often considered a domesticated species, although populations frequently occur naturally. This species contributed US$5.26 billion to the value of alfalfa hay in 2009 (Calderone 2012). In addition to managed bees, there are more than 20,000 species of wild bees in the world, many of which contribute to crop pollination, and all of them contribute to pollination of wild flowers.

There are few long-term population-level studies involving bumblebees and other bee species, and in many cases, the impacts of pesticide exposure and dosage are unclear. These species differ from honeybees in that they generally exhibit smaller foraging ranges and often prefer to nest in the ground. Therefore, populations located near agricultural operations and associated pesticide applications may have fewer alternative options for food and habitat resources. Furthermore, ground-nesting species may face additional exposure risks (i.e. pesticide-contaminated soil) that are not encountered by honeybees, but which remain to be evaluated. Finally, whilst bumblebees tend to be bigger, solitary bees are often smaller than honeybees; thus, these species likely receive a different dose relative to their body weight than honeybees do.

Likely levels of exposure of wild bee species are poorly understood. Whilst neonicotinoid levels have been quantified in the nectar and pollen of various crop plant species (Cresswell 2011; Anon 2012), the degree to which wild bees utilize these resources has not been measured, and furthermore, basic values of toxicity, such as LD_50_ and LC_50_, are completely lacking for the vast majority of these species. The few studies that do exist have employed a range of methods with conflicting results so that drawing general conclusions is difficult at this stage. Moreover, these studies are criticised for low sample size, which limits power to detect effects and/or highly unnatural laboratory conditions.

It is clear that neonicotinoids and fipronil are highly toxic to all bee species tested so far, which in addition to honeybees includes various *Bombus* species, several social stingless bee species and the solitary species *O. lignaria* and *M. rotundata* (Scott-Dupree et al. 2009; Valdovinos-Núñez et al. 2009; Gradish et al. 2010; Mommaerts et al. 2010; Tomé et al. 2012). Cresswell et al. (2012a, b) demonstrated that bumblebees exhibit sublethal responses to imidacloprid at 10 ppb, whilst honeybees were unaffected at this concentration. Scott-Dupree et al. (2009) found that *O. lignaria* is more sensitive to both clothianidin and imidacloprid than *Bombus impatiens*, with *M. rotundata* more sensitive still. Stark et al. (1995) found no difference in the 24 h contact LD_50_ of imidacloprid between honeybees and the solitary bee species *M. rotundata* and *N. melanderi*. Scott-Dupree et al. (2009) demonstrated that *B. impatiens* individuals were more tolerant of thiamethoxam and clothianidin than *O. lignaria* and *M. rotundata*. However, the orchard bee *O. lignaria* exhibits delayed hatching and development when fed imidacloprid at rates from 30 to 300 μg/kg (Abbott et al. 2008). Arena and Sgolastra (2014) compared the acute toxicity of numerous pesticides and found that *Scaptotrigona postica* and *M. rotundata* were more sensitive than honeybees to fipronil, whilst *N. melanderi* was more tolerant. Together, these results suggest that “other” bees may be at least as sensitive, if not more sensitive, to neonicotinoids than honeybees, although more work is clearly needed.

A number of studies have used queenless micro-colonies of bumblebees (containing only workers) to examine the sublethal effects of cumulative neonicotinoid exposure to low, field-realistic doses. Several have found no detectable effects; for example, Tasei et al. (2000) exposed *Bombus terrestris* micro-colonies to 6–25 ppb of imidacloprid and found no significant response. Similarly, Franklin et al. (2004) exposed *B. impatiens* to concentrations of up to 36 ppb of clothianidin without detecting an impact (see also Morandin and Winston 2003). Most recently, Laycock et al. (2012a, b) exposed micro-colonies of *B. terrestris* to a range of concentrations of imidacloprid (0–125 μg/l) and detected a 30 % reduction in fecundity at doses as low as 1 ppb. In the only comparable work on other bee species, Abbott et al. (2008) injected concentrations of up to 300 ppb of neonicotinoids into pollen stores of *O. lignaria* and *M. rotundata* with no measurable impact on larval development.

Interestingly, negative effects seem to be detected more frequently and at lower concentrations when bees have to forage at a distance, even when the distances are tiny. Mommaerts et al. (2010) found no impact of imidacloprid exposure on micro-colonies of *B. terrestris* at field-realistic concentrations when food was provided in the nest, but when workers had to walk just 20 cm down a tube to gather food they found significant sublethal effects on foraging activity, with a median sublethal effect concentration (EC_50_) of just 3.7 ppb. The same researchers also studied queenright colonies foraging in a glasshouse where food was 3 m from their nest and found that ingestion of 20 ppb of imidacloprid caused significant worker mortality, including bees dying at the feeder. Significant mortality was also observed at 10 ppb, but not at 2 ppb. This may explain why some lab studies have failed to find effects.

With impacts more pronounced when bees have to leave the colony, one might predict more marked effects when bees are foraging naturally, travelling kilometres across the landscape (Knight et al. 2005; Osborne et al. 2008). Only four studies have examined impacts of exposure to neonicotinoids on non-*Apis* bees when free-flying in the landscape. Tasei et al. (2001) placed *Bombus lucorum* colonies in the field for 9 days, either adjacent to an imidacloprid-treated field or a control field of sunflowers. During this time, 54 % more of the foragers from the ten imidacloprid-exposed colonies failed to return compared to the ten control colonies; however, this difference was not statistically significant as sample sizes were very small. After 9 days, the colonies were returned to the lab and fed ad libitum. Treated colonies grew more slowly but the difference was not significant. Gill et al. (2012) provided *B. terrestris* colonies with feeders containing 10 ppb of imidacloprid in sugared water whilst simultaneously allowing bees freedom to forage outside the nest. Bees exposed to imidacloprid brought back pollen less often and tended to bring back smaller loads, compared to control bees. Feltham et al. (2014) simulated exposure of queenright *B. terrestris* colonies to a crop of flowering oilseed rape, providing them with sugared water and pollen containing 0.7 and 6 ppb of imidacloprid, respectively, for 2 weeks. They found a 57 % reduction in the mass of pollen brought back to colonies, which persisted for at least 4 weeks after treatment ceased. Only one study to date has attempted to examine the effects of exposure to neonicotinoids on colony-level development of bumblebees under field conditions; Whitehorn et al. (2012) used the same field-realistic doses as Feltham et al. (2014) and then allowed colonies to develop naturally in the field. They recorded significantly reduced nest growth and an 85 % decrease in queen production in imidacloprid-exposed colonies compared to control colonies. This reduction in colony performance is likely due to a combination of factors such as reduced pollen input (as demonstrated by Gill et al. 2012 and Feltham et al. 2014) and perhaps impaired fecundity of queens (following Laycock et al. 2012a, b). In an 11 week greenhouse study, caged queenright colonies of *B. impatiens* were fed treatments of 0, 10, 20, 50 and 100 ppb of imidacloprid, respectively, and clothianidin in sugar syrup (50 %) (Scholer and Krischik 2014). At 6 weeks, queen mortality was significantly higher in 50 and 100 ppb and by 11 weeks in 20–100 ppb neonicotinyl-treated colonies. Starting at 20 ppb, there is a statistically significant reduction in queen survival (37 % for imidacloprid, 56 % for clothianidin), worker movement, colony consumption and colony weight compared to 0 ppb treatments. At 10 ppb imidacloprid and 50 ppb clothianidin, fewer males were produced (Scholer and Krischik 2014).

Bryden et al. (2013) conceived a model to simulate bumblebee colony development to assess the colony-level impacts of well-known sublethal effects on individuals. Their study shows that bumblebee colonies fail when exposed to sustained sublethal levels of pesticide. This is explained by impairment of colony function. Social bee colonies have a positive density dependence, and they are subject to an Allee effect. There is a critical stress level for the success of a colony such that a small increase in the level of stress can make the difference between failure and success.

It seems likely that intoxicated bees are fully able to gather food when it is presented to them within the nest, but when bees have to navigate over realistic distances to extract nectar and pollen from complex, patchily distributed flowers, the effects of intoxication become evident. Studies have focused mainly on behavioural effects in adult bees shortly after exposure to neonicotinoids, but there is evidence from both honeybees (Yang et al. 2012) and stingless bees (Tomé et al. 2012) that exposure during larval stages can impair development of the central nervous system and, hence, result in reduced adult performance several weeks after colony exposure. Therefore, the implications for risk assessment are clear; lab trials, and even trials where colonies are placed immediately adjacent to treated crops, are not appropriate for detecting these impacts. Similarly, experiments need to run for many weeks to examine the long-term effects of exposure on bee health.

The existing toxicological data suggests that impacts on diverse bee taxa are broadly similar at the level of the individual bee, with some evidence that bumblebees and solitary bees may be more susceptible than honeybees. It is clear that field-realistic doses of neonicotinoids can have a range of significant detrimental effects on larval development, adult fecundity, adult foraging behaviour and colony performance in social species. However, the effects of neonicotinoids on the vast majority of bee species have not been examined, and caution is necessary when extrapolating from social to solitary species. No studies have evaluated the impacts of neonicotinoids on solitary species under field conditions. It might plausibly be argued that the large colony size exhibited by honeybees and some stingless bees could buffer these species against reductions in foraging performance, as well as any navigational errors on the part of workers; however, this is unlikely to be the case for either bumblebee colonies, which have just a few hundred workers at most, or solitary bees, where a single female has sole responsibility for provisioning of offspring. Thus, impacts at the population level may be inversely related to levels of sociality. This possibility awaits experimental investigation.

### Butterflies and moths (Lepidoptera)

Among agricultural practices, pesticide use is known to impact butterflies and moths; however, based on observational field data, it is difficult to distinguish the impacts of pesticides from other agricultural customs, such as fertilizer application or landscape simplification, e.g. by removal of hedgerows (Geiger et al. 2010). In the case of butterflies or moths that inhabit structures adjacent to areas where pesticides are applied via aerial spraying, indirect effects of drift from spraying may pose risks both during and after applications (Sinha et al. 1990). In the 1980s for example, helicopter application of pesticides in vineyards of the Mosel Valley in Germany nearly led to the extinction of an isolated population of the Apollo butterfly (*Parnassius apollo*) which was restricted to adjacent rocky slopes (Kinkler et al. 1987; Richarz et al. 1989; Schmidt 1997). In Northern Italy, butterfly communities in natural grasslands have suffered drastic declines downwind of intensively sprayed orchards, leading to the disappearance of all but the most generalist species (Tarmann 2009). Furthermore, spray applications of pesticides may alter soil quality (Freemark and Boutin 1995) and thereby indirectly affect the larvae and pupae of moth species residing in the upper layers of the soil surface during the spring.

Contrary to other non-target species (e.g. bees, birds, spiders, ground beetles), very few comparative pesticide sensitivity tests have been carried out for butterflies and moths. This is surprising given the significant role these insects play for conservation programs. One such study conducted by Brittain et al. (2010b) evaluated the impact of pesticides on various groups of pollinators. When comparing intensively managed systems (high pesticide application rates) with less intensively managed systems (fewer pesticide applications), the authors demonstrated that fewer bumblebee and butterfly species were observed in intensively managed habitat patches. The study also demonstrated that wild bees have higher pesticide-related risks than butterflies (Brittain et al. 2010b).

Moreover, studies by Feber et al. (1997) and Rundlöf et al. (2008) have demonstrated negative impacts of pesticides on butterflies. Both studies evaluated the impacts of organic versus conventional agriculture on butterfly populations. In each case, organic farms were found to host greater numbers and species of butterflies. This response was likely due in part to reduced applications of herbicides in organic systems, as herbicides reduce the abundance of host and nectar plants that are crucial for the survival of larvae as well as adults (Boggs 2003). In contrast, similar studies comparing Lepidopteran communities between organic and conventional agriculture systems found no differences in the number or species richness of butterflies (Weibull et al. 2000 and Brittain et al. 2010a). In the case of these studies, characteristics of the surrounding landscape such as the absence of specific vegetation elements (e.g. hedgerows or floral nectar sources) at both the local and regional scales, or the broad scale application of pesticides, may have influenced the outcome of the findings.

In contrast to the few ecological and ecotoxicological studies on the direct and indirect impacts of pesticides on non-target Lepidoptera, numerous results are available on the impacts of pesticides on the butterfly and moth species that are regarded as agricultural pests during the larval stage (Haynes 1988; Davis et al. 1991a, b, 1993; Liang et al. 2003). The impacts of systemic pesticides on Lepidoptera have been investigated for some 32 pest species of moths in nine different families (Table 2). This represents a tiny fraction of the estimated 200,000 Lepidoptera species. The results demonstrate considerable variation in the impact of pesticides on different species of Lepidoptera. For example, Doffou et al. (2011a, b) noted that the susceptibility of two cotton pests, *Pectinophora gossypiella* (Gelechiidae) and *Cryptophlebia leucotreta* (Tortricidae), to acetamiprid differs almost 3-fold (LD_50_ = 11,049 and 3,798 ppm, respectively). First instar *Cydia pomonella* caterpillars (Tortricidae) are more than 100 times as sensitive as final fifth instar caterpillars, with an LC_50_/LC_90_ of 0.84/1.83 and 114.78/462.11 ppm, respectively (Stara and Kocourek 2007a, b).

**Table 2:** Studies on the effects of systemic pesticides in Lepidoptera

Family	Species	Host	Imidacloprid	Thiamethoxam	Clothianidin	Acetamiprid	Thiacloprid	Dinotefuran	Fipronil
Gelechiidae	*Pectinophora gossypiella*	Cotton				Doffou et al. (2011a, b)			
Gelechiidae	*Phthorimaea operculella*	Potato	Symington (2003)				Saour (2008)		Dogramaci and Tingey (2008)
Gracillariidae	*Cameraria ohridella*	Horse chestnut tree	Stygar et al. (2013)						
Gracillariidae	*Phyllocnistis citrella*	Citrus	Villanueva-Jimenez and Hoy (1998), Setamou et al. (2010)						
Gracillariidae	*Phyllonorycter ringoniella*	Apple	Funayama and Ohsumi (2007a, b)	Funayama and Ohsumi (2007a, b)	Funayama and Ohsumi (2007a, b)	Funayama and Ohsumi (2007a, b)	Funayama and Ohsumi (2007a, b)	Funayama and Ohsumi (2007a, b)	
Lyonetiidae	*Leucoptera coffeella*	Coffee		Diez-Rodrijguez et al. (2006)					
Noctuidae	*Agrotis ipsilon*	Corn and various crops			Kullik et al. (2011a)				
Noctuidae	*Helicoverpa armigera*	Various crops	Ahmad et al. (2013)						
Noctuidae	*Helicoverpa zea*	Cotton	Kilpatrick et al. (2005)	Kilpatrick et al. (2005)		Kilpatrick et al. (2005)			
Noctuidae	*Heliothis virescens*	Tobacco							Pedibhotla et al. (1999)
Noctuidae	*Lacanobia subjuncta*	Apple and various fruits		Brunner et al. (2005)	Brunner et al. (2005)	Brunner et al. (2005)			
Noctuidae	*Sesamia inferens*	Rice							Fang et al. (2008)
Noctuidae	*Spilarctia obliqua*	Polyphagous	Ansari et al. (2012)						
Noctuidae	*Spodoptera litura*	Polyphagous	Abbas et al. (2012)						Ahmad et al. (2008), Huang et al. (2006a, b)
Psychidae	*Thyridophteryx ephemeraeformis*	Thuja and other ornamental plants			Rhainds and Sadof (2009)			Rhainds and Sadof (2009)	
Pyralidae	*Acrobasis vaccinii*	Blueberry				Wise et al. (2010)	Wise et al. (2010)		
Pyralidae	*Cactoblastis cactorum*	Opuntia	Bloem et al. (2005)						
Pyralidae	*Chilo infuscatellus*	Sugarcane							Mann et al. (2009)
Pyralidae	*Chilo suppressalis*	Rice	Yu et al. (2007a, b)						Fang et al. (2008), He et al. (2013), Chen and Klein (2012), Cheng et al. (2010), He et al. (2007, 2008), Li et al. (2007)
Pyralidae	*Ostrinia nubilalis*	Stored grain	Yue et al. (2003)	Yu et al. (2007a, b)					Durham et al. (2001, 2002), Siegfried et al. (1999)
Pyralidae	*Plodia interpunctella*	Stored grain	Yue et al. (2003)	Yue et al. (2003)					
Pyralidae	*Tryporyza incertulas*	Rice	Wang et al. (2005)						
Sesiidae	*Pennisetia marginata*	Raspberry	McKern et al. (2007)						
Tortricidae	*Choristoneura rosaceana*	Apple		Brunner et al. (2005)	Brunner et al. (2005)	Brunner et al. (2005), Dunley et al. (2006)			
Tortricidae	*Cryptophlebia leucotreta*	Cotton				Doffou et al. (2011a, b)			
Tortricidae	*Cydia pomonella*	Apple		Brunner et al. (2005)	Brunner et al. (2005)	Brunner et al. (2005), Cichon et al. (2013), Knight (2010), Magalhaes and Walgenbach (2011), Mota-Sanchez et al. (2008)	Cichon et al. (2013), Magalhaes and Walgenbach (2011), Stara and Kocourek (2007), Voudouris et al. (2011), Reyes et al. (2007)		
Tortricidae	*Epiphyas postvittana*	Trees	Taverner et al. (2012)				Taverner et al. (2011, 2012)		
Tortricidae	*Grapholita lobarzewskii*	Apples	Charmillot et al. (2007)				Charmillot et al. (2007)		
Tortricidae	*Grapholita molesta*	Apple		Jones et al. (2012)		Magalhaes and Walgenbach (2011), Jones et al. (2010)	Magalhaes and Walgenbach (2011)		
Tortricidae	*Pandemis pyrusana*	Apple		Brunner et al. (2005)	Brunner et al. (2005)	Brunner et al. (2005), Dunley et al. (2006)			
Tortricidae	*Rhyacionia frustrana*	Pine trees	Asaro and Creighton (2011a, b)						Asaro and Creighton (2011)
Yponomeutidae	*Plutella xylostella*	Cabbage	Hill and Foster (2000)			Ninsin et al. (2000a, b), Sayyed and Crickmore (2007), Ninsin and Tanaka (2005), Ninsin (2004a, b), Ninsin and Miyata (2003)			Li et al. (2006), Sayyed and Wright (2004), Shi et al. (2004), Zhou et al. (2011)

Not surprisingly, different neonicotinoid compounds vary in toxicity. Thiacloprid and acetamiprid for example are recorded to have stronger effects on the survival of *Phyllonorycter ringoniella* than all other neonicotinoid substances (Funayama and Ohsumi 2007a, b). Acetamiprid has been shown to be more toxic than thiacloprid in several studies, but the degree of difference varies greatly. For example, a study by Cichon et al. (2013) found thiacloprid to be two times as toxic to *C. pomonella* as acetamiprid (LC_99_/LC_50_ = 1.55/0.17 vs 0.71/0.08 ppm, respectively), whilst Magalhaes and Walgenbach (2011) recorded a 60-fold difference in the sensitivity of the same species to these compounds (LC_50_ = 1.06 and 65.63 ppm, respectively).

Many studies have documented systemic pesticide resistance in Lepidoptera; for example, *Phtorimaea operculella* has been found to be resistant to fipronil (Doğramacı and Tingey 2007), *Spodoptera litura* to both fipronil and imidacloprid (Huang et al. 2006a, b; Ahmad et al. 2008; Abbas et al. 2012), *C. pomonella* to acetamiprid and thiacloprid (Cichon et al. 2013; Knight 2010; Stara and Kocourek 2007a, b), and *Plutella xylostella* to acetamiprid (Ninsin et al. 2000a, b). In the latter field study from Japan, an almost 10-fold higher dosage was required to reach the same lethal concentration (LC_50_/_95_ = 315/2,020 compared to 35.1/137 ppm in susceptible laboratory colonies). Applications of such high concentrations may further increase negative impacts on non-target species of insects. Even low sublethal doses can have severe impacts on Lepidoptera populations. In a study on *Helicoverpa armigera* by Ahmad et al. (2013), a 16th of the LC_50_ of imidacloprid (5.38 ppm) increased the next generation survival rate by a factor of 4 (i.e. equivalent to LC_10_) compared to a treatment with the LC_50_ dose. Sublethal effects included a significant reduction in the survival and fecundity as well as increased mortality in the first and subsequent generations. Asaro and Creighton (2011a, b) noted that loblolly pines appeared to be protected from the Nantucket pine tip moth (*Rhyacionia frustrana*) even 1 year after treatment, and the treatment effect apparently was not confined to the target pest species, but extended to further non-target insect species.

There is a clear need for studies on the impact of pesticides on butterflies and moths and in particular those species that are not agricultural pests, but which commonly inhabit managed landscapes. Extensive studies on the direct and indirect effects of pesticides on these non-target groups are urgently needed on different geographical scales and across long time periods (Aebischer 1990) and which include all developmental stages of butterflies and moths (i.e. egg, larva, pupa, adult). It is of paramount importance to include varying intensities of pesticide applications, their persistence and their interplay with biotic and abiotic factors (Longley and Sotherton 1997; Brittain et al. 2010b).

### Other invertebrates

This section will review the studies on neonicotinoids and non-target organisms, in particular the predatory invertebrates of natural pest species. Biological pest control plays an important role in integrated pest management (Byrne and Toscano 2007; Peck and Olmstead 2010; Prabhaker et al. 2011; Khani et al. 2012) with studies suggesting that predators may contribute to the similarity in crop yields between non-treated and pesticide-treated fields (Albajes et al. 2003; Seagraves and Lundgren 2012).

### Routes of exposure

Non-target organisms can be exposed to neonicotinoid pesticides in a variety of ways. Predatory invertebrates may become contaminated by consuming pests such as leafhoppers or aphids that feed on treated crops (Albajes et al. 2003; Papachristos and Milonas 2008; Moser and Obrycki 2009; Prabhaker et al. 2011; Khani et al. 2012). Direct contamination through the diet can also be a problem for other beneficial plant-feeding invertebrates (Dilling et al. 2009; Girolami et al. 2009; Moser and Obrycki 2009; Prabhaker et al. 2011; Khani et al. 2012). For example, several species of hoverfly and parasitoid wasps attack agricultural pests, but also subsidise their diet with nectar. Therefore, these insects can be affected by neonicotinoids, which are translocated into the nectar and pollen of treated crop plants (Stapel et al. 2000; Krischik et al. 2007).

Other routes of exposure include contact with treated surfaces, exposure to sprays or consumption of guttation droplets (Papachristos and Milonas 2008; Prabhaker et al. 2011; Khani et al. 2012). For example, neonicotinoid soil drenches or injections have been found to adversely affect the development of Lepidoptera larvae pupating within the soil (Dilling et al. 2009), whilst soil drenches have been found to significantly lower the overall abundance of insect species and species richness. In one study, imidacloprid was used on eastern hemlock (*Tsuga canadensis*) to effectively control the hemlock woolly adelgid (*Adelges tsugae*); however, the abundance of non-target detrivorous, fungivorous and phytophagous invertebrates was significantly lower in soil drench and injection treatments, when compared to untreated plots (Dilling et al. 2009).

Parasitoid wasps such as *Gonatocerus ashmeadi* can come into contact with neonicotinoids when emerging from the eggs of its host. One such host, the glassy-winged sharpshooter (*Homalodisca itripennis*), a common agricultural pest of many different crops, lays its eggs on the underside of leaves, beneath the epidermal layer. If eggs are laid on neonicotinoid-treated plants, *G. ashmeadi* nymphs may be exposed to toxins when they emerge from the egg and chew through the leaf to get to the surface (Byrne and Toscano 2007).

A 3 year study by Peck (2009) found that when imidacloprid was used as a lawn treatment to target neonate white grubs (Coleoptera: Scarabaeidae), it exhibited cumulative detrimental effects on the abundance of Hexapods, Collembola, Thysanoptera and Coleoptera adults, which were suppressed by 54–62 % overall throughout the course of the study. Population numbers of non-target organisms can also be indirectly affected by a reduction in prey or host species (Byrne and Toscano 2007; Dilling et al. 2009).

### Diptera

Of the Diptera, the genus *Drosophila* provides well-known and convenient model species for toxicity testing. Mechanisms of resistance to imidacloprid and its metabolism have been studied in *Drosophila melanogaster*. Particularly, cytochrome P450 monooxygenases (CYPs) are involved, as is the case in mosquitoes (Riaz et al. 2013). According to Kalajdzic et al. (2012), three P450 genes (Cyp4p2, Cyp6a2 and Cyp6g1) located on the 2R chromosome were highly up-regulated in imidacloprid-resistant flies. However, the same authors did not find that imidacloprid induced expression of Cyp6g1 and Cyp6a2 (Kalajdzic et al. 2013). More recently, it has been shown that imidacloprid was metabolized to eight derivatives in *D. melanogaster*. In this process, only the P450 Cyp6g1 was involved in the enhanced metabolism in vivo (Hoi et al. 2014). Direct toxicity (LC_50_) has been determined for various *D. melanogaster* strains. For instance, the toxicity of several neonicotinoids and butene-fipronil was evaluated (Arain et al. 2014) with neonicotinoids being less toxic than butene-fipronil. It was suggested that differences exist between adults and larvae. Acute LC_50_ values can be compared to LC_50_ measured after chronic exposure, within two studies. With a mutant strain, Frantzios et al. (2008) found a decrease by a factor of 2 for adult flies (acute vs chronic) and a factor of 3 for larvae. Very recently, Charpentier and co-workers have distinguished between male and female flies, from a field strain (Charpentier et al. 2014). Here, the chronic LC_50_ was 29 times lower than the acute LC_50_ for males; it was 172 times lower for females and 52 times lower for larvae. Additionally, this study demonstrated that a significant increase of mortality (27–28 %), with a V-shape, was occurring at concentrations 1,100 and 4,600 times lower than the chronic LC_50_ for males and females, respectively. Other parameters that are crucial for reproduction were tested (mating and fecundity). The LOEC was determined at a concentration that is 3,300,000 and more than 7,900,000 times lower than the acute LC_50_ for females and males, respectively. These data can be linked to data concerning mortalities observed by chronic exposure of bees at very low concentrations.

### Hymenoptera (excluding bees)

A few studies have investigated the effect of neonicotinoid pesticides on parasitic wasps used as biological control agents. Stapel et al. (2000) found that the parasitoid wasp *Microplitis croceipes* had significantly reduced foraging ability and longevity after feeding on extrafloral nectar of cotton (*Gossypium hirsutum*) treated with imidacloprid. Prabhaker et al. (2007) give acute toxicity for two different exposure times for the parasitic wasp species *Eretmocerus eremicus*, *Encarsia formosa*, *Aphytis melinus* and *G. ashmeadi* (Table 3).

**Table 3:** Acute neonicotinoid toxicity for different Hymenoptera species (Prabhaker et al*.* 2007)

Species	48 h exposure time mg (AI)/ml		24 h exposure time mg (AI)/ml
	Acetemiprid	Thiamethoxam	Imidacloprid
*Eretmocerus eremicus*	108.27	1.01	1.93
*Encarsia formosa*	12.02	0.397	0.980
*Gonatocerus ashmeadi*	0.134	1.44	2.63
*Aphytis melinus*	0.005	0.105 (24 h exposure time)	0.246

In another study, *Anagyrus pseudococci* (a nectar-feeding wasp) was fed using buckwheat (*Fagopyrum esculentum*) flowers that had been exposed to imidacloprid as a soil treatment, applied at the label rate. Only 38 % of the wasps survived after 1 day, compared to 98 % fed on untreated flowers. This decreased to 0 % survivorship after 7 days for treated flowers, compared to 57 % on the untreated flowers (Krischik et al. 2007).

As stated in the section on exposure routes, exposure to imidacloprid did not affect mortality of *G. ashmeadi* (a parasitoid wasp) during development within its host, and the adults were sensitive during emergence from the host egg. When mortality was assessed within 48 h of emergence, the LC_50_ for the parasitoid was 66 ng of imidacloprid per cm^2^ leaf (Byrne and Toscano 2007).

Neonicotinoids are commonly used in household products as highly concentrated bait formulations to control ants (Rust et al. 2004; Jeschke et al. 2010); however, the use of agrochemical products at less concentrated doses is an issue for non-target ants. For the leafcutter ant *Acromyrmex subterraneus subterraneu*s, Galvanho et al. (2013) found that sublethal doses of imidacloprid reduced grooming behaviour. Grooming behaviour in this ant is a defence against pathogenic fungi like *Beauveria* species. Barbieri et al. (2013) recently discovered that interactions between different ant species may be negatively affected using sublethal doses of neonicotinoids. In interspecific interactions, individuals of a native ant species (*Monomorium antarcticum*) lowered their aggression towards an invasive ant species (*Linepithema humile*) although survival was not affected. Exposed individuals of *L. humile* displayed an increase in aggression with the outcome that the probability of survival was reduced.

### Hemiptera

Whilst many Hemiptera are acknowledged as being problematic agricultural pests, a number are important predators of these pests, although they do also feed on some plant tissues, which would be contaminated by neonicotinoids (Prabhaker et al. 2011). Table 4 shows LC_50_ rates for different Hemiptera species.

**Table 4:** LC_50_ rates for different Hemiptera species

Species	Chemical	LC_50_ residual contact (mg AI/l)		
		Nymphs	Adults	Reference
*Orius Laevigatus*	Imidacloprid	0.04	0.3	Delbeke et al*.* (1997)
*Hyaliodes vitripennis*	Thiacloprid	1.5	0.3	Bostanian et al*.* (2005)
*Hyaliodes vitripennis*	Thiamethoxam	1.43	0.5	Bostanian et al*.* (2005)
*Geocoris punctipes*	Imidacloprid		5.180	Prabhaker et al*.* (2011)
	Thiamethoxam		2.170	
*Orius insidiosus*	Imidacloprid		2.780	
	Thiamethoxam		1.670	

### Neuroptera

It is not only the agricultural use of neonicotinoids that affects beneficial invertebrates. In one study, Marathon 1 % G, a product for amateur use on flowers containing imidacloprid, had been found to affect lacewings (*Chrysopa* spp.) when used at the label rate. Survival rates on untreated flowers were found to be 79 % for adults, compared to 14 % on treated flowers over a 10 day period (Rogers et al. 2007).

### Coleoptera

A number of studies have looked into the effects of neonicotinoids on various taxa of Coleoptera such as Histeridae (Hister beetles) (Kunkel et al. 1999), Carabidae (ground beetles) (Kunkel et al. 2001; Mullin et al. 2010) and Coccinellidae (ladybird beetles) (Smith and Krischick 1999; Youn et al. 2003; Lucas et al. 2004; Papachristos and Milonas 2008; Moser and Obrycki 2009; Eisenback et al. 2010; Khani et al. 2012).

Some Coleoptera, notably in the carabid and staphyliniid families, are voracious predators and are a vital aspect of integrated pest management. For example, although the provision of beetle banks as nesting habitat takes land out of crop production, in wheat (*Triticum aestivum*) fields, any losses have been found to be more than offset by savings from a reduced need for aphid-controlling pesticides (Landis et al. 2000).

Many of these beetle groups are undergoing rapid declines. In the UK, three quarters of carabid species have reduced in numbers, half of which have been undergoing population declines of more than 30 %, although the reason for these considerable declines are unknown (Brooks et al. 2012). These groups have been particularly useful as bioindicators, due to their sensitivity to habitat changes especially in agricultural environments (Kromp 1999; Lee et al. 2001). In the EU Draft Assessment Report for imidacloprid, acute toxicity tests were undertaken on the carabid beetle *Poecilus cupreus*, finding the larvae to be highly sensitive. Despite the rapporteur Member State deeming that the concentrations tested were too high for it to conclude no risk to carabids for use on sugar beet, there was no indication of further research required (EFSA 2006).

When exposed to turf plots treated with imidacloprid, the carabid beetle *Harpalus pennsylvanicus* displayed a range of neurotoxic problems including paralysis, impaired walking and excessive grooming. These abnormal behaviours then rendered the individuals vulnerable to predation from ants (Kunkel et al. 2001). A study by Mullin et al. (2010) exposed 18 different carabid species to corn seedlings treated to field-relevant doses of either imidacloprid, thiamethoxam or clothianidin. Nearly 100 % mortality was observed for all species over 4 days.

Coccinellids predators are well known for their ability to control common pests, both in agricultural and domestic environments. In soil treatments of imidacloprid, reduced mobility and delayed reproduction have been found in pollen-feeding species such as *Coleomegilla maculata* (Smith and Krischick 1999), whilst egg production and oviposition periods of the Mealybug destroyer (*Cryptolaemus montrouzieri*) (Khani et al. 2012) and *Hippodamia undecimnotata* (Papachristos and Milonas 2008) were significantly reduced. Table 5 shows available acute toxicity for some coccinellid species.

**Table 5:** Acute neonicotinoid toxicity for different Coccinellid species

Species	Chemical	LD_50_ (ng AI per beetle)	LC_50_ (μg AI/ml)	Reference
*Sasajiscymnus tsugae*	Imidacloprid	0.71		Eisenback et al*.* (2010)
*Harmonia axyridis*	Imidacloprid		364	Youn et al*.* (2003)
*Harmonia variegata*	Thiamethoxam		788.55	Rahmani et al*.* (2013)
*Cryptolaemus montrouzieri*	Imidacloprid		17.25–23.9	Khani et al*.* (2012)
*Coccinella undecimpunctata*	Imidacloprid		34.2	Ahmad et al*.* (2011)
*Coccinella undecimpunctata*	Acetamiprid		93.5	Ahmad et al*.* (2011)
*Coleomegilla maculata*—adult	Imidacloprid	0.074		Lucas et al*.* (2004)
*Coleomegilla maculata*—larvae	Imidacloprid	0.034		Lucas et al*.* (2004)

*Harmonia axyridis* (harlequin ladybird) larvae were exposed to corn seedlings grown from seeds treated with the label recommended doses of either thiamethoxam or clothianidin. Seventy-two percent of the larvae exhibited neurotoxic symptoms such as trembling, paralysis and loss of coordination, with only 7 % recovery from the poisoning (Moser and Obrycki 2009).

### Arachnida

In addition to crop protection, applications of neonicotinoid insecticides in veterinary medicine have expanded. Imidacloprid is applied to domestic pets as a spot-on formulation against ear mites (*Otodectes cynotis*) (Jeschke et al. 2010). However, studies on mites have found a positive effect on population numbers. Zeng and Wang (2010) found that sublethal doses of imidacloprid (determined for the green peach aphid (*Myzus persicae*)) significantly increased the hatch rate of eggs and pre-adult survivorship of the carmine spider mite (*Tetranychus cinnabarinus*). James and Price (2002) also found that imidacloprid increased egg production by 23–26 % in two-spotted spider mites (*Tetranychus urticae*) in the laboratory. Another study found that fecundity of this species was slightly elevated when treated with thiamethoxam (Smith et al. 2013).

Szczepaniec et al. (2013) discovered that the application of neonicotinoids supressed expression of plant defence genes when applied to cotton and tomato plants. These genes alter the levels of phytohormones and decrease the plant’s resistance to spider mites (*T. urticae*). When mites were added to the crops, population growth increased from 30 to over 100 % on neonicotinoid-treated plants in the greenhouse and up to 200 % in the field experiment. This study was prompted after the same author had investigated an outbreak of *T. urticae* in New York City, USA. In an attempt to eradicate the emerald ash borer beetle (*Agrillus planipennis*) from Central Park, imidacloprid was applied to trees as a soil drench and trunk injections. This resulted in an outbreak of *T. urticae* on elms due to the natural predators being poisoned through ingestion of prey exposed to imidacloprid, combined with fecundity elevation in the mites themselves (Szczepaniec et al. 2011).

Another study found that thiamethoxam and imidacloprid treatments significantly increased two-spotted spider mite (*T. urticae*) densities on cotton plants when compared to the untreated controls (Smith et al. 2013). This study suggested that the increased usage of neonicotinoids could explain the recent infestation increases of two-spotted spider mite occurring in various crops across the mid-south of the USA.

### Earthworms (Lumbricidae)

Earthworms are vitally important members of the soil fauna, especially in agricultural soils where they can constitute up to 80 % of total soil animal biomass (Luo et al. 1999). They play critical roles in the development and maintenance of soil physical, chemical and biological properties (Lee 1985). Their activities improve soil structure by increasing porosity and aeration, facilitating the formation of aggregates and reducing compaction (Edwards and Bohlen 1996; Mostert et al. 2000). Soil fertility is enhanced by earthworm effects on biogeochemical cycling (Coleman and Ingham 1988; Bartlett et al. 2010), the modification of microbial biomass and activity (Sheehan et al. 2008), breakdown of plant litter (Knollengberg et al. 1985) and the mixing of litter with soil (Wang et al. 2012a).

Neonicotinoid and other systemic insecticides can pose a risk of harm to earthworm survival and behaviour, potentially disrupting soil development and maintenance processes. The same neural pathways that allow neonicotinoids to act against invertebrate pests (Elbert et al. 1991) are also present in earthworms (Volkov et al. 2007). Thus, when neonicotinoids are applied for the protection of agricultural and horticultural crops, earthworms can be exposed by direct contact with the applied granules or seeds, or with contaminated soil or water. Moreover, their feeding activities may result in ingestion of contaminated soil and organic particles (e.g. Wang et al. 2012b). Foliar residues in plant litter after systemic uptake from soils or from direct plant injections also pose a risk to litter-feeding earthworms that consume the contaminated plant litter (e.g. Kreutzweiser et al. 2009).

Neonicotinoids can persist and move in soils thereby increasing the likelihood that earthworms will be exposed for extended periods of time. Laboratory and field trials with neonicotinoids have demonstrated that their half-life in soils varies depending on soil conditions but can range from several weeks to several years (Cox et al. 1997; Sarkar et al. 2001; Cox et al. 2004; Bonmatin et al. 2005; Fossen 2006; Gupta and Gajbhiye 2007; Goulson 2003). Imidacloprid is the most widely used neonicotinoid, and its adsorption to soils is increased by moisture and organic matter content (Broznic et al. 2012), resulting in increased imidacloprid concentrations in organic-rich soils compared to low-organic soils (Knoepp et al. 2012). Earthworms generally prefer moist, organic-rich soils. When soil organic carbon content is low, the high solubility of imidacloprid renders it mobile and it is readily moved through soils (Broznic et al. 2012; Knoepp et al. 2012; Kurwadkar et al. 2013), thereby increasing the likelihood that earthworms could be exposed to the pesticide in soils outside the direct area of application.

#### Effects on survival

Neonicotinoids can be highly toxic to earthworms. However, reported median lethal concentrations (LC_50_) were variable depending on the particular insecticide, test conditions, route of exposure and duration (Table 6). In 13 separate studies, the reported LC_50_ ranged from 1.5 to 25.5 ppm, with a mean of 5.8 and median of 3.7 ppm. In seven studies that reported lowest concentrations at which effects on survival were measureable, those lowest effective concentrations ranged from 0.7 to 25 ppm, with a mean of 4.7 and median of 1.0 ppm. *Eisenia fetida* was the most common test species in these survival studies and represented the range of reported lethal concentrations, giving little indication from among these studies that other species were more sensitive than *E. fetida*.

**Table 6:** Impacts of neonicotinoids and fipronil on earthworms. The impact rating scale is as follows: −−, large decrease; −, moderate decrease; 0, little or no measurable effect (where little is either a small or a brief change); +, moderate increase; and ++, large increase. Endpoints are listed together, separated by a semi-colon, for studies that examined multiple measurement endpoints. Lowest effective concentration is the lowest concentration at which a significant effect was reported, not necessarily the mathematically modelled lowest effective concentration

Taxa	Insecticides	Location	Measurement endpoint	Impact	LC/EC_50_	Lowest effective concentration	Reference
*Eisenia fetida*	Imidacloprid	China	Contact toxicity survival; soil toxicity survival	−; −	LC_50_ = 0.027 μg cm^−2^; LC_50_ = 2.82 ppm		Wang et al*.* (2012a)
*Eisenia fetida*	Imidacloprid	France	Survival; biochemical (hsp70); avoidance	−; −; ++		0.66; 0.66; 0.2 ppm	Dittbrenner et al*.* (2012)
*Eisenia fetida*	Imidacloprid	France	Survival; body mass	−; −		0.66; 0.2 ppm	Dittbrenner et al*.* (2011a)
*Eisenia fetida*	Imidacloprid	UK	Cocoon production; weight change	−; −−	EC_50_ = 1.41; EC_50_ = 2.77 ppm		Gomez-Eyles et al*.* (2009)
*Eisenia fetida*	Imidacloprid	China	Survival	−	LC_50_ = 2.30 ppm		Zang et al*.* (2000)
*Eisenia fetida*	Imidacloprid	China	Survival	−	LC_50_ = 2.30 ppm	1 ppm	Luo et al*.* (1999)
*Eisenia fetida*	Imidacloprid	Canada	Survival; weight loss	−; −−		25; 14 ppm	Kreutzweiser et al*.* (2008b)
*Eisenia fetida*	Fipronil	Brazil	Survival; reproduction; avoidance	0; −; +		>1,000; 62; >10 ppm	Alves et al*.* (2013)
*Eisenia fetida*	Clothianidin	China	Contact toxicity survival; soil toxicity survival	−; −−	LC_50_ = 0.28 μg cm^−2^; LC_50_ = 6.06 ppm		Wang et al*.* (2012b)
*Eisenia fetida*	Thiacloprid	China	Contact toxicity survival; soil toxicity survival	−; −−	LC_50_ = 0.45 μg cm^−2^; LC_50_ = 10.96 ppm		Wang et al*.* (2012a)
*Eisenia fetida*	Thiacloprid	UK	Cocoon production; weight change	−; −−	EC_50_ = 0.968; EC_50_ = 19.0 ppm	0.291; 1.91 ppm	Gomez-Eyles et al*.* (2009)
*Eisenia fetida*	Acetamiprid	China	Contact toxicity survival; soil toxicity survival	−; −−	LC_50_ = 0.0088 μg cm^−2^; LC_50_ = 1.52 ppm		Wang et al*.* (2012a)
*Eisenia fetida*	Nitenpyram	China	Contact toxicity survival; soil toxicity survival	−; −−	LC_50_ = 0.22 μg cm^−2^; LC_50_ = 3.91 ppm		Wang et al*.* (2012a)
*Lumbricus terrestris*	Imidacloprid	France	Survival; biochemical (hsp70); avoidance	0; +; 0		4 ppm	Dittbrenner et al*.* (2012)
*Lumbricus terrestris*	Imidacloprid	France	Survival; body mass	0; −		2 ppm	Dittbrenner et al*.* (2011b)
*Lumbricus terrestris*	Imidacloprid	USA	Feeding activity; abundance	−; −		43 mg m^−2^	Tu et al*.* (2011)
*Lumbricus terrestris*	Imidacloprid	France	Burrowing	−		2 ppm	Dittbrenner et al*.* (2011b)
*Lumbricus terrestris*	Imidacloprid	France	Body mass change; cast production	−; −−	NA; EC_50_ = 0.84 ppm	0.66; 0.66 ppm	Dittbrenner et al*.* (2010)
*Lumbricus terrestris*	Imidacloprid	France	Cast production; body mass change	−; −	LC_50_ = 10.7 ppm	1.89; 0.189 ppm	Capowiez et al*.* (2010)
*Lumbricus rubellus*	Imidacloprid and thiacloprid mixture	UK	Survival; weight change; cocoon production; metabolism	0; −; −−; 0	EC_50_ imidacloprid = 1.46 and EC_50_ thiacloprid = 1.28 ppm		Baylay et al*.* (2012)
*Aporrectodea caliginosa*	Imidacloprid	France	Survival; biochemical (hsp70); avoidance	0; −; ++		2; 2 ppm	Dittbrenner et al*.* (2012)
*Aporrectodea caliginosa*	Imidacloprid	France	Survival; body mass	−; −−		2; 0.66 ppm	Dittbrenner et al*.* (2011a)
*Aporrectodea caliginosa*	Imidacloprid	France	Burrowing	−		0.2 ppm	Dittbrenner et al*.* (2011b)
*Aporrectodea caliginosa*	Imidacloprid	France	Body mass change; cast production	−; −−	NA; EC_50_ = 0.76 ppm	0.66; 0.66 ppm	Dittbrenner et al*.* (2010)
*Aporrectodea nocturna*	Imidacloprid	France	Weight loss; avoidance; burrowing	−; +; −		0.5; 0.1; 0.05 ppm	Capowiez and Berard (2006)
*Aporrectodea nocturna*	Imidacloprid	France	Burrowing	−		0.1 ppm	Capowiez et al*.* (2006)
*Aporrectodea nocturna*	Imidacloprid	France	Survival, weight loss	−; −	LC_50_ = 3.74 ppm	0.1 ppm	Capowiez et al*.* (2005)
*Aporrectodea nocturna*	Imidacloprid	France	Burrowing	−		0.01 ppm	Capowiez et al*.* (2003)
*Allolobophora icterica*	Imidacloprid	France	Weight loss; avoidance; burrowing	−; +; −−		0.5; 0.01; 0.05 ppm	Capowiez and Berard (2006)
*Allolobophora icterica*	Imidacloprid	France	Burrowing	−		0.1 ppm	Capowiez et al*.* (2006)
*Allolobophora icterica*	Imidacloprid	France	Survival, weight loss	−; −−	LC_50_ = 2.81 ppm	0.1 ppm	Capowiez et al*.* (2005)
*Allolobophora icterica*	Imidacloprid	France	Burrowing	−		0.01 ppm	Capowiez et al*.* (2003)
*Dendrobaena octaedra*	Imidacloprid	Canada	Survival; leaf decomposition	0; −		31 ppm	Kreutzweiser et al*.* (2009)
*Dendrobaena octaedra*	Imidacloprid	Canada	Survival; weight loss; reproduction; leaf decomposition	−; −−; −; −	LC_50_ = 5.7 ppm	3; 3; 7; 7 ppm	Kreutzweiser et al*.* (2008b)
*Dendrobaena octaedra*	Imidacloprid	Canada	Survival; weight loss; reproduction; leaf decomposition	0; −; 0; −		11; 3.2 ppm	Kreutzweiser et al*.* (2008a)
*Eisenia andrei*	Imidacloprid	Brazil	Survival; reproduction; avoidance	−; −−; ++	LC_50_ = 25.53; EC_50_ = 4.07; EC_50_ = 0.11 mg/kg	25; 0.75; 0.13 ppm	Alves et al*.* (2013)
Pheretima group	Imidacloprid	South Africa	Survival	−	LC_50_ = 3.0 ppm		Mostert et al*.* (2002)
Pheretima group	Fipronil	South Africa	Survival	0		>300 ppm	Mostert et al*.* (2002)
*Apporectodea* spp*.*	Clothianidin	USA	Abundance; biomass; cast production	−; −; −	NA, field applications		Larson et al*.* (2012)

When compared to other common insecticides, neonicotinoids tend to be among the most toxic to earthworms. Wang et al. (2012a) tested the acute toxicities of 24 insecticides to *E. fetida* and found that the neonicotinoids were the most toxic in soil bioassays and that acetamiprid and imidacloprid in particular were the two most toxic insecticides overall. They also reported that a contact toxicity bioassay demonstrated that the neonicotinoids were extremely toxic by a contact route of exposure (LC_50_ of 0.0088 to 0.45 μg cm^−2^), although the units of contact toxicity concentration were difficult to compare to standard lethal concentrations. Across a broader range of 45 pesticides, Wang et al. (2012b) found that in soil bioasssays, the neonicotinoid insecticide, clothianidin, was the most toxic pesticide to *E. fetida*. Alves et al. (2013) compared three insecticides used for seed treatment and reported that imidacloprid was the most toxic to earthworms. In soil bioassays with five different insecticides, Mostert et al. (2002) found that imidacloprid was the second most toxic (behind carbaryl) to earthworms. We found only two studies that reported the toxicity of fipronil, another common, agricultural systemic insecticide, and both found it to be substantially (at least 100 times) less lethal to earthworms than the neonicotinoids (Mostert et al. 2002; Alves et al. 2013).

#### Effects on reproduction

Only a few studies tested sublethal effects of neonicotinoids on earthworm reproduction, but it is apparent that reductions in fecundity can occur at low concentrations (Table 6). Baylay et al. (2012) reported EC_50_s for imidacloprid and thiacloprid against cocoon production by *Lumbricus rubellus* of 1.5 and 1.3 ppm, respectively, whilst Gomez-Eyles et al. (2009) found similar EC_50_s for the same two insecticides at 1.4 and 0.9 ppm for *E. fetida*. The latter study also reported measurable reductions in cocoon production at 0.3 ppm of thiacloprid. Alves et al. (2013) reported an EC_50_ for reproduction effects of imidacloprid on *Eisenia andrei* of 4 ppm with measureable adverse effects at 0.7 ppm. Kreutzweiser et al. (2008b) tested the effects of imidacloprid in forest litter on the litter-dwelling earthworm *Dendrobaena octaedra* and reported significant reductions in cocoon production among surviving earthworms at 7 ppm.

#### Effects on behaviour

A number of studies focused on behavioural endpoints under the premise that effects on behaviour are often ultimately linked to population or community effects (Little 1990; Dittbrenner et al. 2012). The behavioural attributes considered here are avoidance behaviour, burrowing, cast production and weight change (as an indicator of feeding behaviour). Among the 31 reported values for behavioural effects, weight change was the most common, followed by burrowing, avoidance behaviour and cast production (Table 6). Only a few studies gave median effective concentrations (EC_50_), and they ranged from 0.1 (avoidance) to 19 (weight change) ppm, with a mean EC_50_ of 3.7 and median of 1.3 ppm. These behavioural EC_50_s were about 1.5 to 2.8 times lower than the mean and median lethal concentrations of 5.8 and 3.7 ppm.

However, many more studies reported lowest concentrations at which behavioural effects were detected, and those ranged from 0.01 to 14 ppm with a mean of 1.2 and median of 0.5 ppm. Thus, measurable behavioural effects were more sensitive endpoints than measurable survival effects. Measurable behavioural effects occurred at concentrations of about two to four times lower than the mean and median lowest effective concentrations on survival of 4.7 and 1.0 ppm. Burrowing (smaller, shorter, more narrow burrows) was the most sensitive behavioural endpoint with effects detected at mean and median concentrations of 0.3 and 0.07 ppm (range 0.01 to 2, *n* = 8). Avoidance behaviour was the next most sensitive endpoint with effects detected at mean and median concentrations of 0.5 and 0.13 ppm (*n* = 5), followed by cast production (mean 1.1, median 0.7 ppm, *n* = 3) and weight change (mean 2.1, median 0.7 ppm, *n* = 13). All of these indicate that measurable adverse effects on earthworm behaviour would be expected at neonicotinoid concentrations below 1 ppm in soil.

#### Risks to earthworms

The actual risk of harmful effects on earthworm populations posed by neonicotinoid insecticides will depend on exposure concentration, exposure duration, route of exposure, rate of uptake and inherent species sensitivity. From the toxicity studies reviewed here, it appears that individual earthworms across all common species are at risk of mortality if they consume soil or organic particles with neonicotinoid insecticide concentrations of about 1 ppm or higher for several days. Higher numbers (up to 50 %) of earthworms would be expected to be at risk of mortality when concentrations reach about 3 ppm and higher. Although it was difficult to compare the exposure concentrations to standard bioassays, it appears that the risk of mortality from surface contact exposure can be ten times or more higher than the risk of mortality from consumption of contaminated soils (Wang et al. 2012a). On the other hand, the route of exposure can affect the likelihood of lethal effects on earthworms. When earthworms were exposed to foliar residues in leaf litter from imidacloprid-injected trees, a significant feeding inhibition effect was detected that reduced leaf consumption but did not cause earthworm mortality, even at concentrations of about 10 ppm (Kreutzweiser et al. 2008a).

The risk of sublethal effects on some important behavioural attributes is higher than the risk of mortality to individuals. Insecticide effects on burrowing and avoidance behaviours would be expected at concentrations of about 0.1 to 0.5 ppm and higher. Whilst alterations in burrowing behaviour, especially reductions in burrowing depths, have implications for the transfer properties of soils (Capowiez et al. 2006; Dittbrenner et al. 2011b), the consequences in real-world field conditions are not clear. Fewer, smaller and shorter burrows could reduce air, water and solute transport through soils affecting overall soil ecology, but none of the studies we found actually tested these implications in experimental or field settings.

The concentrations that pose risk of mortality (assuming high toxicity by contact exposure) and sublethal effects on earthworms fall within the range of reported field concentrations, albeit at the upper end of that range of concentrations. Dittbrenner et al. (2011b) indicate that predicted environmental concentrations for imidacloprid in agricultural soils would be about 0.3 to 0.7 ppm, suggesting risks of at least sublethal effects on earthworms could be quite high. Bonmatin et al. (2005) reported that imidacloprid in soils can reach several hundred parts per billion shortly after sowing of treated seeds. Soil samples from a tea plantation treated with clothianidin had average concentrations of up to 0.45 ppm shortly after application (Chowdhury et al. 2012). Donnarumma et al. (2011) found concentrations of imidacloprid in soils at about 0.6 to 0.8 ppm by 2 weeks after application of treated seeds. Ramasubramanian (2013) reported clothianidin concentrations in soils of 0.27 to 0.44 ppm up to 3 days after single applications and 0.51 to 0.88 ppm by 3 days after double applications of water-soluble granules. Collectively, these studies show that operational applications of neonicotinoids can result in soil concentrations that are likely to pose a high risk of sublethal effects and potential risk of lethal effects (especially by contact toxicity) to earthworms.

At least two issues related to the assessment of risk to earthworms from exposure to neonicotinoids have not been adequately addressed in the published literature. The first is the length of exposure periods in toxicity testing compared to the length of exposure to persistent concentrations in natural soils. Most toxicity tests are short term, in the order of days to weeks. On the other hand, neonicotinoid residues can persist in soils for months to years (Bonmatin et al. 2014, this issue). For most pesticides, lethal or effective concentrations become lower as exposure periods increase, and this is likely the case for neonicotinoids (Tennekes 2010; Tennekes and Sánchez-Bayo 2012, 2013; Rondeau et al. 2014). It is plausible that long-term low-level concentrations of neonicotinoids in soils may pose higher risk to earthworms than what can be inferred from the published toxicity tests. The second issue pertains to the heterogeneous distribution of neonicotinoid residues in natural soils. When residues enter the soil at the surface from spray or granule deposition or from litter fall, concentrations in soils are likely to be higher on or near the surface than in deeper soils. Residues entering soils from planted seed or from contaminated water are likely to be higher at or near the source of contamination than elsewhere. Both situations would result in concentration “hot spots” near the points of entry. Conversely, most toxicity tests prepare test concentrations as parts per million (or equivalent) and assume complete mixing. Therefore, levels of exposure to earthworms at or near those hot spots in natural soils will consequently be higher than would be predicted from residue analyses of bulk samples from laboratory or field test systems.

Mortality or behavioural effects on individual earthworms do not necessarily translate to population effects with ecological consequences. Populations of organisms with short generation times (e.g. several generations per year as is the case for most earthworm species) and/or high dispersal capacity have a higher likelihood of recovery from pesticide-induced population declines than those with longer regeneration periods and limited dispersal capacity (Kreutzweiser and Sibley 2013). However, the tendency for neonicotinoids to persist in organic soils reduces the likelihood of this recovery pathway because subsequent generations may be exposed to concentrations similar to those to which the parent generation was exposed. Life history strategies and their influences on community responses and recovery from pesticide effects have been demonstrated by population modelling of other non-target organisms (Wang and Grimm 2010), and similar principles may apply to assessing risks to overall earthworm populations and communities. Population models that account for differential demographics and population growth rates within communities have been shown to provide more accurate assessments of potential pesticide impacts on populations and communities than conventional lethal concentration estimates can provide (Stark and Banks 2003). The use of ecological models to incorporate a suite of factors including seasonal variations, community assemblage mechanisms and lethal and sublethal insecticide effects and their influences on the risks to organisms, populations or communities can provide useful insights into receptor/pesticide interactions and can thereby improve risk assessments (Bartlett et al. 2010). Ecological and population modelling combined with pesticide exposure modelling and case-based reasoning (drawing on past experience or information from similar chemical exposures) can provide further refinements and improve risk assessment for earthworm communities and their ecological function (van den Brink et al. 2002). Empirical field studies of earthworm population responses to realistic field concentrations of neonicotinoids are lacking and would greatly improve risk assessment efforts.

## Aquatic invertebrates

### Freshwater invertebrates

Aquatic invertebrates are extremely important components of aquatic ecosystems. They play roles as decomposers, grazers, sediment feeders, parasites and predators. They also provide much of the food that vertebrates associated with these systems feed upon. Pesticides, including neonicotinoids, reach surface waters through various routes, but in particular through atmospheric deposition (drift) after application by various sprayers, by surface runoff and by seepage of contaminated groundwater. Aquatic invertebrates are particularly susceptible to pesticides. Unlike terrestrial organisms, aquatic organisms generally cannot avoid exposure easily by moving to uncontaminated areas, particularly when pesticides are water soluble. Uptake of pesticides in aquatic invertebrates occurs through respiration (gills and trachea), feeding and through the epidermis, be it cuticle or skin.

Neonicotinoids have been used for a comparatively shorter period of time than other insecticides. However, they are found in freshwater systems more and more frequently. For example, surface water monitoring for pesticides in California has revealed that imidacloprid has frequently exceeded water quality guidelines of 1 ppb (Starner and Goh 2012). In the Washington State, USA, the State Department of Ecology and the State Department of Agriculture have been monitoring salmon-bearing rivers and streams for pesticides, including imidacloprid for a number of years and this insecticide is frequently found (http://agr.wa.gov/PestFert/natresources/SWM/).

However, even though imidacloprid and other neonicotinoids are present in freshwater systems, the question remains to what extent such concentrations affect aquatic organisms in the field. Here we discuss a number of studies dealing with neonicotinoid toxicity to aquatic invertebrates and make some observations about their potential impact on aquatic ecosystems.

#### Laboratory studies

##### Crustacea and Amphipoda

Several laboratory studies have been published on the toxicity of the neonicotinoid imidacloprid on a range of aquatic invertebrates (Table 7). Stark and Banks (2003) developed acute toxicity data and population-level toxicity data for the water flea *Daphnia pulex* exposed to thiamethoxam (Actara). Thiamethoxam was the least toxic insecticide evaluated in this study of seven insecticides, and its LC_50_ of 41 ppm was well above any anticipated concentration expected to be found in surface water systems.

**Table 7:** Selection of studies on the effects of imidacloprid on freshwater macrophauna

	Compound	Experimental design	Effect	LC_50_/EC_50_	LOAEL	Reference
Aquatic taxa						
Oligochaeta	Imidacloprid	10 day exposure to contaminated sediment	Survival, growth, behaviour, avoidance		<0.05 mg/kg	Sardo and Soares (2010)
*Chironomus tentans* and *Hyalella azteca*	Imidacloprid	Standard toxicity test	Survival	0.91 μg/l (28 days)		Stoughton et al*.* (2008)
Mesocosm communities	Neonics and other insecticides		Drift response			Berghahn et al*.* (2012)
*Daphnia*, *Gammarus pulex*	Imidacloprid		Survival			Ashauer et al*.* (2011)
Mayflies	Imidacloprid		Nymph abundance emergence patterns and adult body size			Alexander et al. (2008)
*Ceriodaphnia dubia*	Imidacloprid	Lab toxicity tests	Mortality Population growth rate	2.1 ppb		Chen et al. (2010)
*D. magna*	Imidacloprid	Lab toxicity tests	Mortality	10.4 mg/l		Song et al. (1997)
*Aedes aegypti*	Imidacloprid	Lab toxicity tests	Mortality	44 ppb		Song et al. (1997)
*Aedes taeniorhynchus*		Lab toxicity tests	Mortality	13 ppb		Song et al. (1997)
Mayflies, Oligochaetes	Imidacloprid		Feeding inhibition			Alexander et al. (2008)
Odonata, Libellulidae	Imidacloprid, fipronil	Field	Larval and adult survival, emergence			Jinguji et al. (2013)
Macro-invertebrate community	Imidacloprid	Stream mesocosm	Community diversity, leaf litter breakdown			Pestana et al. (2009)
Crustacean: *Asellus aquaticus*, *Gammarus fossarum*	Imidacloprid and atrazine	Standard toxicity test	Survival, respiration, electron transport system			Lukancic et al. (2010)
Caddisfly: *Seriocostoma*, *Chironomis ripartus*	Imidacloprid	Standard toxicity test	Burrowing behaviour; antipredator behaviour			Pestana et al. (2009)
Ostracoda, *Daphnia magna*	Imidacloprid	Lab toxicity test	Survival			Sánchez-Bayo (2006)
*Chironomus diutus*	Imidacloprid + mixtures (chlorpyrifos, dimethoate)	Lab toxicity test	Survival			LeBlanc et al. (2012)
Terrestrial taxa						
Aphidius ervi	Imidacloprid + cadmium	Lab toxicity tests	Population growth rate			Kramarz and Stark (2003)

Chen et al. (2010) estimated the acute toxicity of imidacloprid to the water flea, *Ceriodaphnia dubia* (LC_50_ = 2.1 ppb), and the chronic toxicity to *C. dubia* populations. The effects of the adjuvant, R-11 alone and in combination with imidacloprid were also assessed. In the population study, exposure of *C. dubia* to imidacloprid concentrations of 0.3 ppb reduced population size to 19 % of the control population. This concentration is well below the U.S. EPA’s expected environmental concentration of 17.4 ppb, indicating that imidacloprid may cause damage to aquatic invertebrates in the field.

The acute and chronic effects of imidacloprid on the amphipod *Gammarus pulex* were studied by Nyman et al. (2013). Feeding by *G. pulex* and body lipid content were significantly reduced after exposure to a constant imidacloprid concentration of 15 ppb. Furthermore, *G. pulex* individuals were unable to move and feed after 14 days of constant exposure resulting in a high level of mortality.

Interestingly, the standard test organism *Daphnia magna* is especially insensitive to neonicotinoids (Beketov and Liess 2008). An acute LC_50_ of around 7,000 ppb is several orders of magnitude above effective concentrations found for several other invertebrates. This implies that *D. magna* cannot be used as a sensitive test organism protective for many species.

##### Insecta

Acute toxicity estimates of neonicotinoids on aquatic insects have also been published. LC_50_ estimates for aquatic insects range from 3 to 13 ppb. Imidacloprid LC_50_ estimates for the mayfly *Baetis rhodani*, the black fly *Simulium latigonium* (Beketov and Liess 2008) and the mosquito *Aedes taeniorhynchus* (Song et al. 1997) are 8.5, 3.7 and 13 ppb, respectively. LC_50_ estimates for *B. rhodani* and *S. latigonium* exposed to thiacloprid were 4.6 and 3.7 ppb, respectively (Beketov and Liess 2008). A chronic LC_50_ of 0.91 ppb was reported for the midge *Chironomus tentans* for imidacloprid (Stoughton et al*.* 2008). A study on the effects of imidacloprid as a mixture with the organophosphate insecticides dimethoate and chlorpyrifos on the midge *Chironomus dilutus* found that imidacloprid acted synergistically with chlorpyrifos and antagonistically with dimethoate (LeBlanc et al. 2012).

##### Oligochaetes

Sardo and Soares (2010) investigated the effects of imidacloprid on the aquatic oligochaete *Lumbriculus variegatus*. They exposed this worm species to imidacloprid concentrations ranging from 0.05 to 5.0 mg/kg sediment. Mortality was fairly low (35 % in the highest concentration), but *L. variegatus* avoided imidacloprid-contaminated sediment. Furthermore, individual growth (biomass) was inhibited at all concentrations tested compared to the control.

##### Mesocosm studies

Alexander et al. (2008) examined the effect of imidacloprid as a 12 day pulse or 20 day continuous exposure on the mayflies *Epeorus* spp. and *Baetis* spp. Nymph densities were reduced after both types of exposures. Sublethal effects were observed as well. Adults were smaller and had smaller head and thorax size after exposure to imidacloprid concentrations as low as 0.1 ppb. However, these effects were only found in males.

Within community test systems, neonicotinoids had strong effects especially on insects (Hayasaka et al. 2012). However, to our knowledge, all experiments investigating a dose–response relationship observed effects at the lowest concentrations evaluated. Hence, it is difficult to establish a NOEC. Within outdoor mesocosm studies, a LOEC of 1.63 ppb was estimated for imidacloprid. Adverse effects on benthic communities with 5 % reductions in the abundance of invertebrates were observed by Pestana et al. (2009). For thiacloprid, strong effects on sensitive long living insects were observed at pulsed exposure to 0.1 ppb (Liess and Beketov 2011), the lowest effective concentration observed so far in communities.

Berghahn et al. (2012) conducted stream mesocosm studies whereby 12 h pulses of imidacloprid (12 ppb) were introduced three times at weekly intervals. Results showed that drift of insects and the amphipod *Gammarus roeseli* increased after exposure to pulses of imidacloprid. These results indicated that imidacloprid was having a negative effect on *G. roeseli*.

In another stream mesocosm study, Böttger et al. (2013) evaluated pulses of imidacloprid on *G. roeseli*. The number of brood carrying females was reduced in the imidacloprid treatments compared to the control groups in the last 3 weeks of the study.

The populations of an aquatic invertebrate, the common mosquito *Culex pipiens*, exposed over several generations to repeated pulses of low concentrations of the neonicotinoid thiacloprid, continuously declined and did not recover in the presence of a less sensitive competing species, the water flea *D. magna*. By contrast, in the absence of a competitor, insecticide effects on the more sensitive species were only observed at concentrations one order of magnitude higher, and the species recovered more rapidly after a contamination event. The authors conclude that repeated toxicant pulse of populations that are challenged with interspecific competition may result in a multigenerational culmination of low-dose effects (Liess et al. 2013).

##### Risk to aquatic ecosystems

A species sensitivity distribution (SSD) of acute toxicity data for a wider range of species, including ostracods, cladocerans and other aquatic organisms, predicts a hazardous concentration for 5 % of aquatic species (HC5) for imidacloprid in water in the range 1.04–2.54 ppb (Sanchez-Bayo and Kouchi 2012).

Van Dijk et al. (2013) developed a regression analysis for abundance of aquatic macro-invertebrate species and nearby imidacloprid concentrations in Dutch surface waters. Data from 8 years of nationwide monitoring covering 7,380 different locations of macro-invertebrate samples and 801 different locations of imidacloprid samples were pooled. Next, the biological samples (macro-invertebrate abundance counts) were combined with nearby (in space and time) chemical samples (imidacloprid concentrations), and next, a statistical analysis was done on the complete pooled combined dataset. They found that macro-invertebrate abundance consistently declines along the gradient of increasing median nearby imidacloprid concentration in the pooled dataset. This pattern turned out to be robust: it is independent of year and location. Overall, a significant negative relationship (*P* < 0.001) was found between abundance of all macro-invertebrate species pooled and nearby imidacloprid concentration. A significant negative relationship was also found for abundance of each of the pooled orders Amphipoda, Basommatophora, Diptera, Ephemeroptera and Isopoda, and for several species separately. The order Odonata had a negative relationship very close to the significance threshold of 0.05 (*P* = 0.051). In accordance with previous research, a positive relationship between abundance and nearby imidacloprid pollution was found for the order Actinedida. However, other pesticides were not included into the analyses by Van Dijk et al. (2013). Therefore, possible co-linearity or synergisms between neonicotinoids and other pollutants still need to be further explored (Vijver and Van den Brink 2014).

Pesticide exposure was identified to strongly reduce the amount and abundance of vulnerable invertebrate species in streams using the SPEAR approach (Liess and von der Ohe 2005). The approach was extended from German streams to Australian, Danish, French and Finnish streams revealing the same effects of pesticide exposure on vulnerable invertebrate species (Rasmussen et al. 2013; Liess et al. 2008; Schäfer et al. 2012). Beketov et al. (2013) analysed the effect of pesticide presence on invertebrate species richness in European (Germany and France) and Australian streams. They found an overall reduction of 42 % for Europe and 27 % for Australia in species richness between uncontaminated and heavily contaminated streams. The limitation of these studies in the context of assessment of neonicotinoid impact is that toxicity was mainly due to insecticides, other than neonicotinoids, as general usage of the latter only increased recently.

The results of laboratory and semi-field (mesocosm) studies indicate that aquatic invertebrates are very sensitive to the neonicotinoid insecticides. However, most of the studies we found in the literature were conducted with imidacloprid. For pesticide risk assessment, the published results to date indicate that it may be difficult to predict community-level effects using the tiered aquatic effect assessment scheme and acute and chronic toxicity data. When extrapolating from acute and chronic single species test systems, the assessment factors identified by the uniform principle of the relevant EU legislation (1107/2009) do not predict safe concentrations in multi-species outdoor mesocosms. For example, acute laboratory effects of thiacloprid on sensitive insect species show that effects occur after exposure to the range of 3–13 ppb. Accordingly, an assessment factor of 100 would indicate a safe concentration of 0.03 to 0.13 ppb for thiacloprid. However, outdoor mesocosm results employing a pulsed exposure show a LOEC below 0.1 ppb for thiacloprid (Liess and Beketov 2011). Lower concentrations were not investigated. Obviously, an assessment factor higher than 100 is needed to identify safe concentrations on the basis of acute test results. For the HC5 calculated on acute lethal concentrations, an assessment factor of larger than 10 is necessary (Liess and Beketov 2012). Additionally, in a laboratory study, chronic effects of sensitive insect species were exhibited after exposure to 0.91 ppb imidacloprid. Employing an assessment factor of 10 would indicate a safe concentration of approximately 0.1 ppb imidacloprid. However, this concentration is not safe according to the results obtained in complex community investigations. Unfortunately, to the best of our knowledge, no community-level investigation with imidacloprid evaluating a range of concentrations below 0.1 ppb has been published. This type of study would help with determining a NOEC for imidacloprid. Overall, the results of the published literature indicate that certain neonicotinoids have the potential to cause significant damage to aquatic ecosystems by causing negative effects in individuals and populations of aquatic invertebrates at very low concentrations. Protective concentrations for these products in aquatic systems still need to be determined.

### Marine and coastal invertebrates

There is very limited information regarding the assessment of the environmental toxicology and contamination of neonicotinoids in marine ecosystems. Standardised environmental toxicological characterization focuses on only a few species models and rarely examines species that represent keystone organisms in marine or coastal ecosystems (CCME 2007). Monitoring and surveillance of neonicotinoid pollution in marine coastal habitats are non-existent.

#### Toxicology

The earliest published marine ecotoxicological studies of neonicotinoids were with opossum shrimps (*Mysidopsis bahia*) which are distributed in marine coastal waters (Ward 1990, 1991; Lintott^ 1992^). Median LC_50_ (96 h) for the technical grade of imidacloprid was 34.1 ppb with a mortality-NOEC of 13.3 ppb (Ward 1990). Exposure to a commercial formulation (ADMIRE) of imidacloprid resulted in a 96 h mortality-NOEC of 21 ppb. Maximum acceptable toxicant concentrations for *M. bahia* to imidacloprid were 23 parts per trillion (ppt) for growth effects and 643 ppt for reproductive effects (Ward 1991).

Toxicology for other marine arthropods includes *Artemia* spp. and a brackish water mosquito (*Aedes taeniohynchus*). The 48 h LC_50_ for *Artemia* was 361 ppm, whilst *Aedes* exhibited a 72 h LC_50_ of 21 ppb, and a 48 h LC_50_ of 13 ppb for an early instar stage of development (Song et al. 1997; Song and Brown 1998). Osterberg et al. (2012) demonstrated that in the blue crab (*Callinectes sapidus*), megalopae were an order of magnitude more sensitive than juveniles to lethal effects of imidacloprid (24 h-LC_50_ = 10 ppb for megalopae vs 24 h-LC_50_ = 1,1 ppb for juveniles).

There are no known published OECD/EPA parameter-based studies on non-arthropod marine invertebrates. For the marine mussel, *Mytilus galloprovincialis*, a transcriptomic and proteomic survey was conducted as a response to imidacloprid and thiacloprid exposures (Dondero et al. 2010). This study concluded that the two neonicotinoids induced distinct toxicodynamic responses and that caution should be heeded when conducting ecological risk assessments for chemical mixtures that target the same receptor. Rodrick (2008) demonstrated that imidacloprid had an effect on oyster hemocyte immunocompetence and that there was an additive effect when oysters were exposed to a compound stress of salinity and exposure to imidacloprid. Tomizawa et al. (2008) used the gastropod *Aplysia californica* as a model to characterize imidacloprid and thiacloprid as agonists of the acetylcholine-binding protein, indicating that neonicotinoids could also affect marine gastropods.

#### Environmental pollution

There are no published works regarding the marine environmental contamination of neonicotinoids. Until recently, there has been little public concern of neonicotinoid non-point source pollution of marine environments from land runoff. At least within the USA, this attitude is beginning to change. In the State of Washington 2013, the Willapa-Grays Harbor Oyster Growers Association received a conditional registration from the U.S. Environmental Protection Agency to use imidacloprid to control native burrowing shrimp in Willapa Bay, Washington that may threaten commercial shellfish beds (EPA Reg. no. 88867–1). In Hawaii, there have been public protests and scrutiny over the use of neonicotinoid pesticides in their industrial agricultural practices and their likely negative impacts on coral reefs and sea grass beds (Sergio 2013). For both Hawaii and the U.S. Virgin Islands, there is concern that the use of neonicotinoids as a method for termite control may be polluting and impacting coastal resources.

## Conclusion

At field-realistic levels of pollution, neonicotinoids and fipronil generally have negative effects on physiology and survival for a wide range of non-target invertebrates in terrestrial, aquatic, marine and benthic habitats. Effects are most often found by in vitro testing, using a limited number of test species. This basically means that there is a deficit of information for the grand majority of other invertebrates. In vitro testing to establish safe environmental concentration thresholds is hindered by the fact that most test protocols are based on older methodology, validated for pesticides with very different chemical and toxicological characteristics. New and improved methodologies are needed to specifically address the unique toxicology of these neurotoxic chemicals, including their non-lethal effects and synergistic effects for a variety of terrestrial, aquatic and marine organisms.

The amount of published in vivo field tests is small and experimental setups often suffer from inability to control for variation in (semi)natural circumstances or have insufficient statistical power due to the high financial costs of large robust field experiments. Given the clear body of evidence presented in this paper showing that existing levels of pollution with neonicotinoids and fipronil resulting from presently authorized uses frequently exceed lowest observed adverse effect concentrations and are thus likely to have large-scale and wide ranging negative biological and ecological impacts, the authors strongly suggest that regulatory agencies apply more precautionary principles and tighten regulations on neonicotinoids and fipronil.
